# Integrated single-cell and spatial transcriptomics reveal divergent immunological and stromal programs in peritoneal versus ovarian endometriosis

**DOI:** 10.1186/s12905-026-04456-5

**Published:** 2026-04-20

**Authors:** Fangjie He, Shuiling Zu, Yan Lin, Jun Shi, Shunhe Lin

**Affiliations:** 1 Department of Gynecology, College of Clinical Medicine for Obstetrics & Gynecology and Pediatrics, Fujian Maternity and Child Health Hospital, Fujian Medical University, Fuzhou, 350000 China; 2 Department of Ultrasound, College of Clinical Medicine for Obstetrics & Gynecology and Pediatrics, Fujian Maternity and Child Health Hospital, Fujian Medical University, Fuzhou, 350000 China

**Keywords:** Endometriosis, Single-cell RNA sequencing, Spatial transcriptomics, Immune-hot niche, Immune-cold niche, CCL19-CCR7 axis, Precision medicine

## Abstract

**Background:**

Endometriosis encompasses heterogeneous lesions, primarily peritoneal and ovarian subtypes, with distinct immunological and fibrotic features. The subtype-specific roles of the immune microenvironment in pathogenesis remain unclear. Integrated analysis combining high-resolution cellular atlases with spatial context is needed to decipher these mechanisms.

**Methods:**

We performed an integrative analysis of published single-cell RNA sequencing (*n* = 81,676 cells) from control endometrium (Ctrl), eutopic endometrium (EuE), peritoneal endometriosis (EcP), and ovarian endometriosis (EcO), and spatial transcriptomic (*n* = 60 segments) data from Ecp and match EuE. Analytical pipelines include cellular atlas construction, differential expression, spatial co-expression, and cell-cell communication inference.

**Results:**

Our single-cell atlas revealed fundamentally distinct cellular ecosystems. EcP was enriched for *CCL19* + perivascular cells (6.4-fold vs. control) and immune-active niches, whereas EcO exhibited pronounced smooth muscle differentiation and NNMT upregulation. Spatial analysis compartmentalized these programs, with the *CCL19*-*CCR7* chemokine axis localized to stromal niches in EcP strongly correlated (ρ = 0.872). In contrast, EcO showed enhanced smooth muscle gene expression (*MYH11*, log₂FC = 3.87). Cell-cell communication networks diverged, with EcP dominated by chemokine and TGF-β signaling, and EcO dominated by smooth muscle and PDGF pathways. Therapeutic target analysis revealed subtype-specific patterns: immune checkpoints (*CTLA4* and *PDCD1*) were upregulated in EcP, whereas *MMP9* was dramatically downregulated in EcO.

**Conclusion:**

EcP and EcO exhibit fundamentally divergent programs. We propose to conceptualize these as an ‘immune-hot’, chemokine-driven inflammatory niche (EcP) versus an ‘immune-cold’, fibromuscular survival niche (EcO). These findings underscore that lesion-specific immune-stromal crosstalk may dictates pathogenesis and suggest a potential paradigm shift toward immunologically-informed, subtype-specific precision therapeutics, which warrant further experimental validation.

**Supplementary Information:**

The online version contains supplementary material available at 10.1186/s12905-026-04456-5.

## Introduction

Endometriosis is a common gynecological disorder affecting approximately 10% of women of reproductive age worldwide, and is characterized by the growth of endometrial-like tissue outside the uterine cavity [1, 2]. It is widely recognized as an immune-mediated condition, where dysregulated local and systemic immune responses contribute to lesion survival, angiogenesis, and pain generation [3, 4]. The disease manifests with debilitating symptoms such as chronic pelvic pain, dysmenorrhea, and infertility, significantly impairing patients’ quality of life [3, 5]. Despite its high prevalence, the pathophysiology of endometriosis remains incompletely understood, posing persistent challenges for early diagnosis and effective treatment [5, 6].

The complexity of this disease partly stems from the remarkable lesion heterogeneity. Peritoneal endometriosis (EcP) and ovarian endometriomas (endometriotic cysts) exhibit distinct clinical presentations, histological architectures, and progression patterns [7]. For instance, EcP is typically superficial and is associated with active inflammatory responses, whereas ovarian endometriosis (EcO) often forms cystic structures characterized by extensive fibrosis [8]. Such histological divergence suggests that different lesion types may harbor unique cellular compositions and molecular regulatory networks.

In recent years, single-cell RNA sequencing (scRNA-seq) has revolutionized our understanding of cellular landscapes within complex tissues [9, 10]. In the field of endometriosis, the seminal work by Tan et al. (2022) established the first comprehensive cellular atlas of the disease by profiling EcP and EcO at single-cell resolution [11]. This study revealed unprecedented cellular diversity, including the discovery of a distinct perivascular cell population expressing *CCL19* and the characterization of immunomodulatory macrophage subsets [11]. An inherent limitation of scRNA-seq is the loss of crucial spatial information during tissue dissociation. The advent of spatial transcriptomics has addressed this gap by enabling gene expression analysis within the preserved morphological context of tissues [12, 13]. Recently, Burns et al. (2025) applied spatial transcriptomics to superficial EcP and identified a pivotal role for the lesion epithelium in driving local inflammation (e.g., via high expression of complement C3) and aberrant crosstalk with macrophages [14]. This study underscored the importance of spatial context in deciphering disease mechanisms.

Recent mechanistic studies have elucidated the specific pathways involved in different lesion contexts [15]. The *CCL19*/*CCR7* axis was shown to activate PI3K/Akt signaling, promoting endometrial stromal cell proliferation and invasion [16], whereas NNMT-mediated anti-apoptotic programs were identified in the EcO epithelium [17]. Comprehensive single-cell profiling has revealed distinct hormonal, immunological, and inflammatory signatures across endometriosis subtypes, with additional evidence of metabolic reprogramming in EcO [18, 19]. Parallel investigations into patient eutopic endometrium (EuE) revealed intrinsic abnormalities including reduced uterine NK cells and impaired stromal decidualization, suggesting that endometriosis originates from “defective seeds” predisposed to ectopic survival [20]. Recent advances in tumor immunology have revealed that the heterogeneity of the tumor microenvironment plays a critical role in regulating disease progression, with molecular subtyping based on immune features enabling precision immunotherapy [21–23]. These findings collectively suggest that different anatomical microenvironments (peritoneal cavity vs. ovarian cortex) may shape lesion fate through distinct microenvironmental cues, as evidenced by studies showing fibroblast heterogeneity and specialized niche interactions in different lesion types [24, 25]. Nonetheless, these studies have primarily focused on single lesion types or specific mechanisms, lacking systematic integrative comparative analyses between EcP and EcO [15]. Consequently, a critical unanswered question remains: how can high-resolution single-cell atlases with spatially contextualized transcriptomic data precisely compare the spatial molecular features, cellular interaction networks, and underlying driving mechanisms between these major endometriosis subtypes?

Therefore, this study aimed to employ an integrative transcriptomic analysis combining single-cell and spatial transcriptomics to investigate and compare the distinct molecular and cellular landscapes of EcP and EcO. Our primary objectives were to: (1) validate and quantify the spatial distribution patterns of key cell populations and their characteristic gene expression signatures within the tissue architecture; (2) systematically identify differential transcriptional programs and cell-cell communication networks between the two lesion subtypes; and (3) explore the implications of these differences in lesion-specific pathophysiology and potential therapeutic targeting. This work is expected to contribute to the elucidation of the molecular basis underlying the complex heterogeneity of endometriosis.

## Methods

### Study design and data sources

This bioinformatics reanalysis systematically investigates endometriosis heterogeneity by integrating published omics data. Our analytical strategy leveraged two complementary public datasets selected for their specific strengths: First, scRNA-seq data were downloaded from GEO under accession number GSE179640 [11]. This dataset, generated by Tan et al., was chosen because it provides the most comprehensive single-cell atlas to date that simultaneously profiles both EcP and EcO, along with the Ctrl and EuE, serving as the essential cellular-resolution foundation for our comparative analysis. Second, spatial transcriptomic data were obtained from GEO under accession number GSE263897 [14]. Generated by Burns et al., this dataset offers high-quality, compartment-annotated (epithelium, stroma and macrophages) spatial data specifically for EcP lesions, with no matched spatial data available for EcO.

. Although it does not include EcO, it is indispensable to address the spatial dimension of our research by (i) validating the spatial niches of EcP-specific cell populations (e.g., *CCL19* + perivascular cells) identified from scRNA-seq and (ii) defining the spatial co-expression patterns and interaction hubs (e.g., *CCL19*-*CCR7* axis) that characterize the organized tissue microenvironment of EcP.

The insights derived from this EcP-specific spatial framework then inform our interpretation of the scRNA-seq-derived differences for EcO, allowing us to hypothesized spatial organization based on single-cell profiles (e.g., smooth muscle differentiation).

We devised a three-phase analytical framework: first, a focused re-analysis of single-cell sequencing data to construct a cellular atlas of lesion cores; second, comparative analysis of spatial transcriptomics segments with single-cell clusters based on compartment-specific annotations and shared gene expression signatures; and finally, quantitative cross-lesion and cross-modal comparative and functional analyses based on this integrated data.

### ScRNA-seq analysis

GSE179640 data were processed using Seurat (v4.3.0) [26]. Quality control filtered cells with < 500 genes, < 1000 unique molecular identifiers (UMIs), or > 25% mitochondrial content. These thresholds were chosen based on standard practices in single-cell RNA sequencing analysis to exclude low-quality cells (low gene/UMI counts) and dying cells (high mitochondrial content), while retaining sufficient cells for downstream analysis [11, 17]. Potential doublets were identified and removed using Scrublet [27]. After filtering, 81,676 high-quality cells were retained for downstream analyses.

Data normalization was performed using the transform method [28]. To integrate cells across samples and correct for batch effects, we applied the harmony algorithm [29]. The effectiveness of batch correction was assessed by visualizing the integration of samples from different sequencing batches in the low-dimensional embedding, which demonstrated co-clustering of cells from different batches within each biological group, indicating successful batch effect removal while preserving biological variation. Dimensionality reduction was carried out via principal component analysis (PCA), followed by visualization in a Uniform Manifold Approximation and Projection (UMAP) embedding based on the top 2000 highly variable genes [30]. Cell clustering was performed using the Leiden algorithm to identify 45 transcriptionally distinct cell populations [31]. The cell populations were annotated based on the expression patterns of canonical marker genes for the epithelial, stromal, endothelial, myeloid, lymphoid, and perivascular lineages.

To focus on the intrinsic features of the lesion core, a critical decision in our comparative analysis was the exclusion of all cells derived from the peritoneal lesion-adjacent tissue samples. Consequently, the primary subjects in this study were divided into four groups: control endometrium (Ctrl), EuE from patients, EcP core, and EcO core.

### Spatial transcriptomics analysis

Spatial transcriptomic data were downloaded from GEO under accession number GSE263897, originating from Burns et al. [14]. Data were generated using the NanoString GeoMx platform and processed using its official pipeline. All 60 spatial segments passed the quality control (gene detection rate > 30%). The gene expression counts were normalized using the Q3 normalization method. This method scales each segment’s expression values based on the 75th percentile of counts, minimizing the influence of technical variation without relying on housekeeping genes. This approach ensures that comparisons across segments and compartments reflect biological differences rather than artifacts of normalization.

Given that each spatial segment was pre-annotated based on fluorescence-activated sorting into three cellular compartments: macrophages (CD68+), epithelium (pan-cytokeratin+), and stroma (ACTA2-, CD68-, pan-cytokeratin-), we analyzed the data at the segment level without additional deconvolution. This compartment-specific annotation allowed the direct comparison of transcriptional programs across biologically defined tissue niches.

For integrative analysis, spatial segments were compared directly to single-cell clusters based on the shared gene expression patterns of compartment-specific markers. Expression matrices from spatial and single-cell datasets were harmonized using gene symbol matching, and comparative analyses focused on genes consistently detected across both platforms (> 5 counts in > 10% cells/spots). This approach enabled the validation of single-cell identified cell states within their spatial context, while maintaining the integrity of the original tissue architecture annotations.

### Differential expression and functional enrichment analysis

Differential expression analyses were performed at two complementary resolutions to balance statistical rigor with biological detail.

For exploratory analysis at single-cell resolution, differential expression was assessed using Welch’s t-test with Benjamini-Hochberg correction on log-normalized expression values. Genes with adjusted *P* < 0.05 and |log₂FC| > 0.25 were considered significant at the cell level for initial gene screening. To account for patient-level biological replication and provide a more conservative statistical framework for core findings, patient-level differential expression analyses were performed using pseudo-bulk analysis as described in Sect.  2.7.

For genes expressed in a limited subset of cells (e.g., CCL19, expressed in < 2% of cells), conventional differential expression analysis comparing all cells between groups can underestimate true biological differences due to dilution by non-expressing cells. To address this, we performed a two-step approach: (i) the proportion of expressing cells (expression > 0) was compared between groups using a two-proportion z-test; (ii) among expressing cells only, the geometric mean of expression levels was calculated and compared using Welch’s t-test on log-transformed values. This approach allows us to distinguish between changes in the frequency of expressing cells and changes in expression intensity within those cells.

Gene-gene correlations were calculated using Spearman’s method on log-normalized expression values across all spatial segments. Hierarchical clustering with complete linkage and Euclidean distance was used to identify co-expression modules. Gene Ontology (GO) enrichment analysis was performed using the clusterProfiler package (v4.0) with Biological Process ontology, applying Benjamini-Hochberg adjustment for multiple testing. Terms with adjusted *P* < 0.05 were considered significant.

### Cell-cell communication inference

Potential cell-cell communication networks were inferred by integrating spatial co-expression patterns with single-cell transcriptional profiles. Spatial co-expression analysis was performed by calculating the Spearman correlation coefficients between the ligand and receptor genes across all spatial transcriptomic segments. Significant correlations (ρ > 0.7, Benjamini-Hochberg adjusted *P* < 0.001) were considered evidence of potential interactions.

Differential expression of signaling components was assessed by comparing the mean expression levels between tissue groups using Welch’s t-test with Benjamini-Hochberg correction on the cell-level scores. For pathway activity analysis, pathway gene sets were manually curated based on literature review and included key biological processes relevant to endometriosis pathophysiology, such as immune response, angiogenesis, smooth muscle contraction, Wnt signaling, hormone response, and extracellular matrix organization. Pathway activity scores were calculated as the average log-normalized expression of genes within each set for each cell, then averaged across cells within each tissue group for comparison.

Signaling networks were constructed by integrating spatial correlation strengths, expression differences, and literature-curated pathway interactions, using the igraph package (v1.3.5). Network nodes represent signaling pathways, with size proportional to the magnitude of differential expression and color indicating lesion subtype specificity.

To account for potential confounding effects from mixed cellular composition within spatial segments, partial correlation analysis was performed to assess the robustness of ligand-receptor associations after controlling for immune cell content. Immune cell score for each spatial segment was calculated as the mean expression of immune marker genes (CD68 for macrophages, CD3D/CD3E for T cells, PTPRC for pan-immune cells, and CD4/CD8A for T cell subsets). Partial Spearman correlations were calculated using the ppcor package in R, and correlations with Benjamini-Hochberg adjusted *P* < 0.05 were considered significant.

### Statistical analysis

All statistical analyses were performed using R version 4.5.2. Sample sizes for single-cell analyses were as follows: Ctrl (*n* = 10,351 cells from three patients), EuE (*n* = 10,806 cells from nine patients), EcP (*n* = 23,251 cells from eight patients), and EcO (*n* = 23,634 cells from four patients), totaling 81,676 high-quality cells from 24 patient-tissue samples. For spatial transcriptomic analyses, all 60 quality-controlled segments from three slides (Slide_1:24 segments, Slide_3:24 segments, and Slide_4:12 segments) were included, comprising 30 EcP segments and 30 EuE segments. Correlation analyses were performed using Spearman’s rank correlation with Benjamini-Hochberg adjustment. Patient-level differential expression was performed using DESeq2 on pseudo-bulk counts as described in Sect.  2.7. Data are presented as the mean ± standard deviation or median with interquartile range, as appropriate.

### Pseudo-bulk validation analysis

To account for patient-level biological replication, we performed pseudo-bulk differential expression analysis. Single-cell counts were aggregated by sample (patient-tissue combination) to generate 24 pseudo-bulk expression profiles (Ctrl: 3, EuE: 9, EcP: 8, EcO: 4 samples). Differential expression analysis was performed using DESeq2 with the design formula ~ group for each pairwise comparison (EcP vs. EcO, EcP vs. Ctrl, EcO vs. Ctrl). Genes with low expression (row sums < 10 across fewer than 3 samples) were filtered out prior to analysis. Genes with |log₂ fold change| > 1 and Benjamini-Hochberg adjusted P-value < 0.05 were considered significantly differentially expressed.

## Results

### Single-cell atlas of endometriosis tissues

**Fig. 1:**
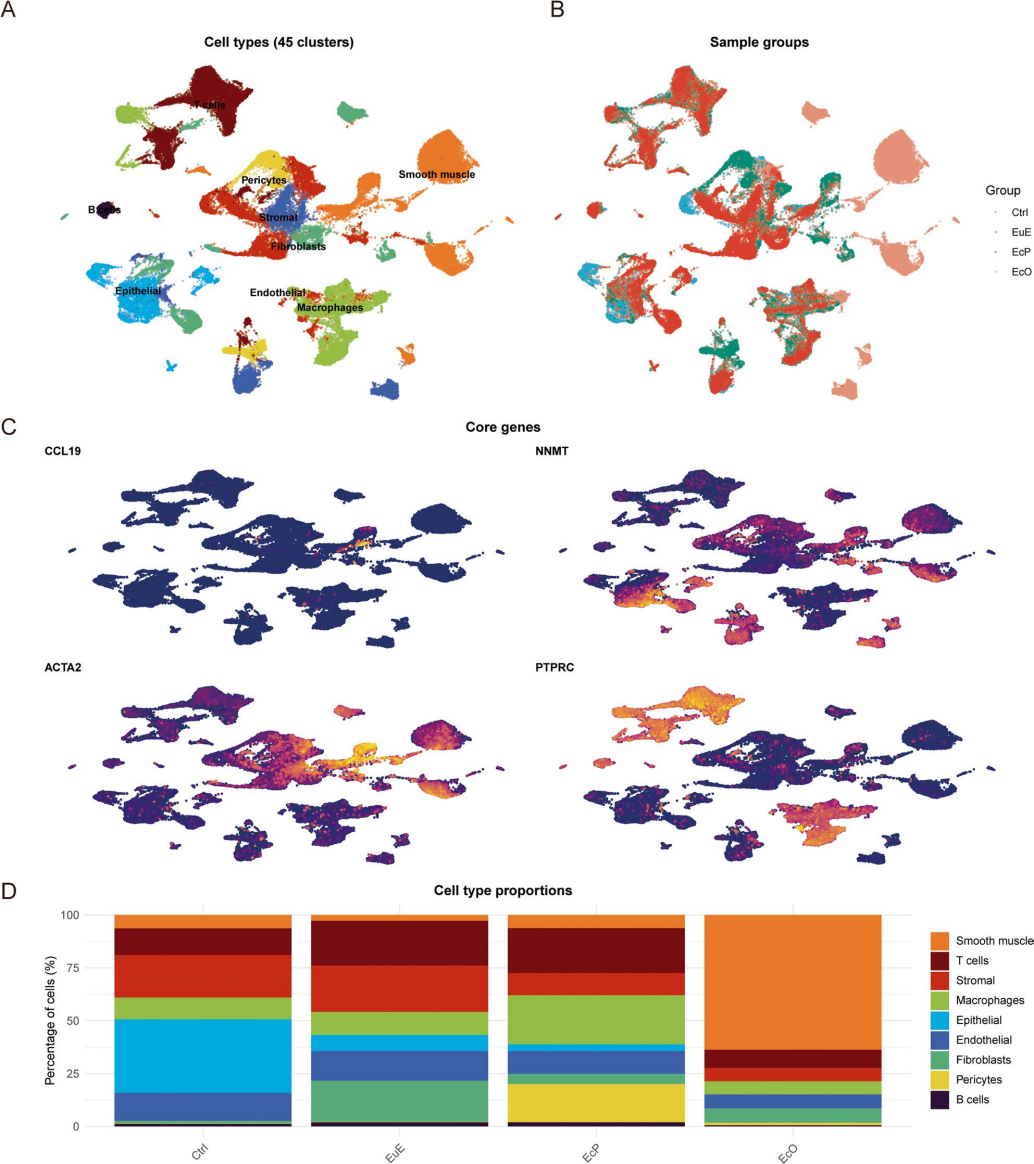
Single-cell atlas of control endometrium (Ctrl), eutopic endometrium (EuE), peritoneal endometriosis (EcP) and ovarian endometriosis (EcO). **A** UMAP visualization of 81,676 single cells colored by cell type annotations. Cell types were assigned based on canonical marker gene expression. **B** UMAP plot showing cells colored by tissue of origin: Ctrl (blue), EuE (red), EcP (green), and EcO (orange). **C** Feature plots displaying expression distribution of core genes: *CCL19* (enriched in EcP stromal populations), *NNMT* (enriched in EcO epithelial subsets), *ACTA2* (smooth muscle marker), and *PTPRC* (pan-immune marker). **D** Cell type proportions across tissue types. Stacked bars show the relative abundance of each cell type in Ctrl, EuE, EcP, and EcO. Note the marked enrichment of smooth muscle cells in EcO and the higher proportions of immune cells (macrophages and T cells) in EcP. Cell types were annotated as described in Fig. 1A and Methods 2.2

Analysis of 81,676 high-quality single cells revealed 45 transcriptionally distinct clusters, capturing comprehensive cellular heterogeneity (Fig. 1A). Quality control metrics, including the number of genes detected, UMI counts, and mitochondrial percentage, confirmed the high quality of the scRNA-seq data across all sample groups (Supplementary Fig. 1A). A strong positive correlation was observed between sequencing depth and gene detection (Pearson *r* = 0.907, *P* = 2.2 × 10⁻¹⁶), indicating technical robustness (Supplementary Fig. 1B).

Cell type identification was performed using canonical marker genes for nine major cell lineages, enabling annotation of all 45 clusters into nine distinct cell types: T cells, macrophages, smooth muscle cells, stromal cells, endothelial cells, fibroblasts, B cells, epithelial cells, and pericytes (Fig. 1A). Marker gene expression patterns confirmed appropriate clustering, with each cell type showing distinct transcriptional signatures. Representative UMAP visualizations of key lineage markers further illustrate the spatial distribution of major cell populations: epithelial cells (*KRT18*), stromal cells (*COL1A1*), macrophages (*CD68*), and T cells (*CD3D*) (Supplementary Fig. 2).

When projected by tissue of origin, cells exhibited a clear separation among the four groups: Ctrl, EuE from patients, EcP, and EcO, indicating distinct global transcriptional profiles across different tissue microenvironments (Fig. 1B). The considerable overlap between EcP and EuE reflects their shared endometrial origin, as EcP lesions derive from ectopic endometrial tissue, while the distinct separation of Ctrl (healthy endometrium) and EcO aligns with the absence of disease-associated alterations and the extensive fibromuscular differentiation of ovarian lesions, respectively. Feature plots of core genes revealed cell type-specific expression patterns: *CCL19* was predominantly expressed in specific stromal populations enriched in EcP, *NNMT* showed selective expression in epithelial subsets, *ACTA2* marked smooth muscle cells, and *PTPRC* identified immune cells (Fig. 1C). Analysis of the cellular composition revealed profound differences in cell type distribution across tissue types (Fig. 1D). EcO was characterized by marked enrichment of smooth muscle cells (39.8% of total cells), whereas EcP showed higher proportions of immune cells, including macrophages and T cells. The Ctrl was enriched for epithelial and stromal cells, while EuE exhibited intermediate proportions of multiple cell lineages. This differential cellular architecture underscores the fundamental heterogeneity between EcP and EcO. Notably, all nine cell types are present across all tissue groups, but their relative proportions vary dramatically, with the most pronounced differences observed for smooth muscle cells (enriched in EcO), immune cells (enriched in EcP), and epithelial/stromal cells (enriched in Ctrl). In the following sections, comparisons between EcP and EcO are used to define subtype-specific features, while EuE is included as a patient-matched reference to distinguish disease-associated alterations from inherent patient characteristics.

### Differential gene expression in endometriosis lesions

**Fig. 2:**
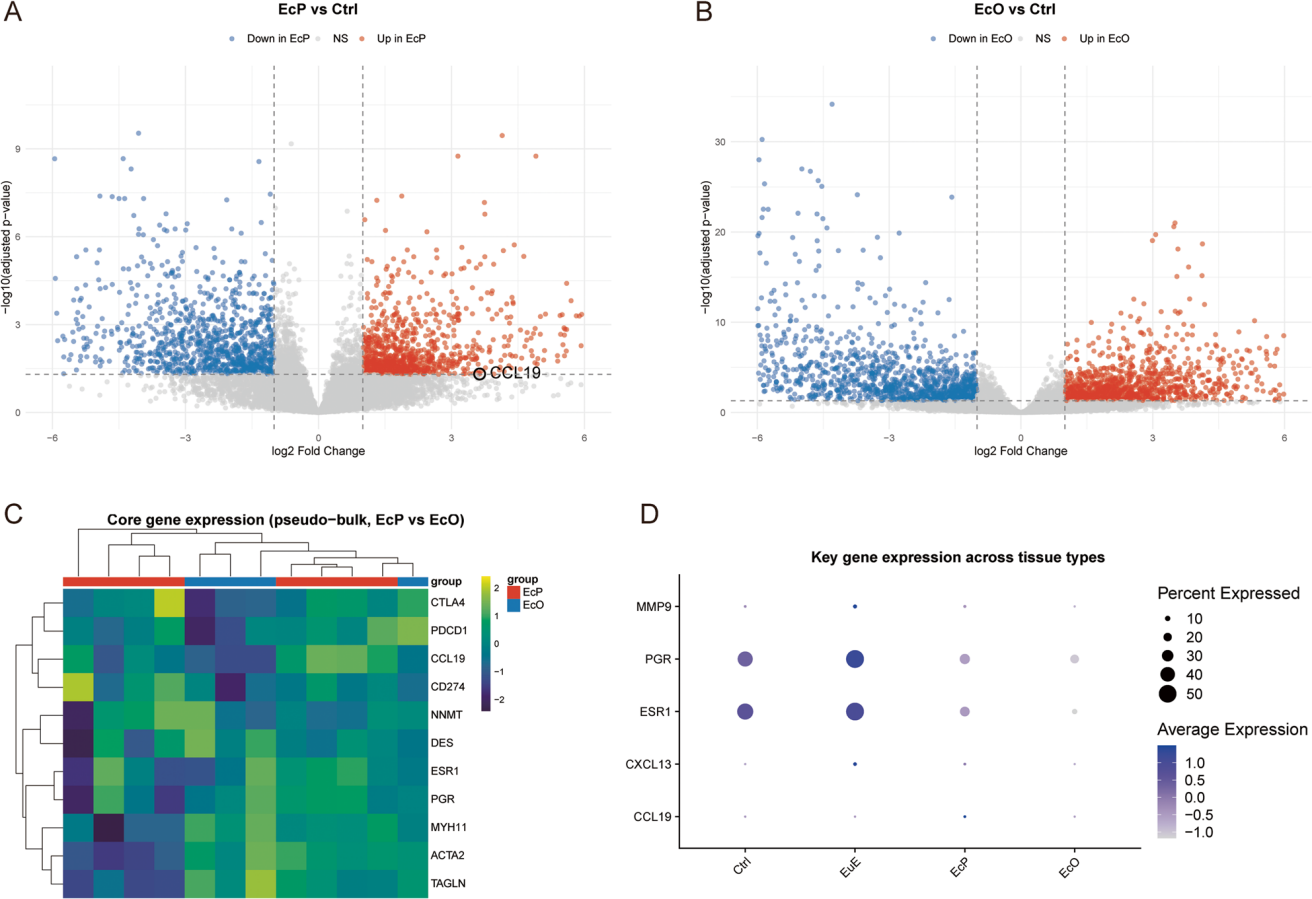
Differential gene expression analysis and pseudo-bulk validation. **A** Volcano plot displaying genes differentially expressed between EcP and Ctrl. Red points indicate significantly upregulated genes (log2FC > 0.5, adjusted *P* < 0.05), blue points indicate downregulated genes, and gray points indicate non-significant genes. The chemokine *CCL19* is highlighted with a black circle. Dashed lines represent significance thresholds (|log₂FC| = 0.5, adjusted *P* = 0.05). **B** Volcano plot for the comparison between EcO and Ctrl, following the same visualization scheme. **C** Heatmap showing pseudo-bulk validated expression of core genes in EcP and EcO samples. Each column represents an individual sample (patient-tissue combination). Expression values are row-scaled Z-scores. Color bars above the heatmap indicate sample groups (EcP: red, EcO: blue). **D** Dot plot showing the expression of key genes across all tissue types (Ctrl, EuE, EcP, EcO). Dot size represents the percentage of expressing cells; color intensity represents the average expression level. *Note: Analysis focused on established lesions; peritoneal lesion-adjacent tissues were excluded*

To establish robust transcriptional profiles that account for inter-patient variability, we first performed patient-level pseudo-bulk differential expression analysis (see Methods 2.7). This analysis revealed distinct transcriptional profiles for EcP and EcO compared with the Ctrl (Fig. 2A-B). (Note: Peritoneal lesion-adjacent tissues were excluded from this comparison to focus on established lesions).

EcP (excluding adjacent tissues) versus the Ctrl demonstrated significant upregulation of smooth muscle and extracellular matrix genes (Fig. 2A). Key findings included: *ACTA2* (α-smooth muscle actin, log₂FC = 2.08, adjusted *P* = 0.002), *FN1* (fibronectin, log₂FC = 1.67, adjusted *P* = 8.11 × 10⁻^5^). The chemokine *CCL19* was specifically enriched in EcP at the patient level (log₂FC = 3.63, adjusted *P* = 0.049, Fig. 2A), confirming its specificity for peritoneal lesions. At the patient level, epithelial marker genes showed downregulation consistent with the reduced proportion of epithelial cells in EcP (Fig. 1D), epithelial marker genes showed downregulation at the transcriptional level. *EPCAM* expression was significantly decreased in EcP compared to Ctrl (log₂FC = -3.49, adjusted *P* = 0.02), while *KRT18* showed a similar trend that did not reach statistical significance (log₂FC = -1.96, adjusted *P* = 0.08).

EcO versus Ctrl exhibited an even more pronounced smooth muscle phenotype (Fig. 2B), with greater upregulation of *MYH11* (log₂FC = 3.87, adjusted *P* = 1.18 × 10⁻^5^), *DES* (log₂FC = 3.59, adjusted *P* = 0.005), and *ACTA2* (log₂FC = 2.68, adjusted *P* = 1.1 × 10⁻^5^). Unlike EcP, EcO showed a trend toward downregulation of angiogenesis-related factors, although VEGFA did not reach statistical significance at the patient level (log₂FC = -0.92, adjusted *P* = 0.43).

Comprehensive validation of the differentially expressed genes confirmed the transcriptional alterations identified in our comparative analyses (Supplementary Fig. 3). Unsupervised hierarchical clustering of differentially expressed genes revealed clear separation by tissue origin, recapitulating the distinct expression profiles observed in Fig. 2 (Supplementary Fig. 3A). Spatial visualization of representative markers on UMAP embeddings confirmed cell type-specific localization patterns, with *MUC5B*,* LYVE1 and VEGFA* enrichment in specific cellular compartments within lesion microenvironments (Supplementary Fig. 3B).

GO enrichment validation confirmed the coordinated activation of biological programs across endometriosis subtypes (Supplementary Fig. 4). Unsupervised hierarchical clustering of key pathway gene sets revealed distinct expression patterns, with immune response and angiogenesis pathways showing elevated activity in EcP, whereas smooth muscle contraction and extracellular matrix organization pathways were more prominently activated in EcO (Supplementary Fig. 4A). Quantitative assessment of pathway activity scores at single-cell resolution demonstrated significant heterogeneity within and between tissue types (Supplementary Fig. 4B). EcP exhibited the highest variance in immune response scores, reflecting considerable intercellular heterogeneity in immune pathway activation within peritoneal lesions. However, the median immune response score in EcP was comparable to Ctrl. In contrast, TGF-β signaling showed both elevated median activity and substantial variance in EcP, suggesting coordinated activation of this pathway in a subset of cells. Spearman correlation analysis among pathways identified strong positive associations between Wnt signaling and extracellular matrix organization (*r* = 0.597) and between smooth muscle contraction and extracellular matrix (ECM) organization (*r* = 0.521), suggesting coordinated regulation of fibrotic and contractile programs in endometriosis pathogenesis (Supplementary Fig. 4C). Spatial visualization of key pathway genes on UMAP embeddings confirmed cell type-specific expression patterns, with representative genes from immune response (*CCL19*), angiogenesis (*VEGFA*), smooth muscle contraction (*ACTA2*), hormone response (*ESR1*), ECM organization (*COL1A1*), and perivascular programs (*RGS5*) showing distinct localization within tissue microenvironments (Supplementary Fig. 4D). These findings validate the pathway enrichment results from our differential expression analysis and reveal the interconnected nature of the biological processes driving endometriosis progression.

### Validation of subtype-specific signatures by pseudo-bulk analysis

To validate the robustness of our findings and exclude the possibility that the observed differences were driven by cell-level pseudoreplication, we performed pseudo-bulk differential expression analysis at the patient level. Single-cell counts were aggregated by sample to generate 24 pseudo-bulk expression profiles, and differential expression was reassessed using DESeq2.

The core findings from our cell-level analysis were consistently reproduced at the patient level (Fig. 2C). CCL19 expression was significantly higher in EcP compared to EcO (log₂FC = 5.75, adjusted *P* = 0.0014) and compared to Ctrl (log₂FC = 3.63, adjusted *P* = 0.049), confirming its specificity for peritoneal lesions. In contrast, CCL19 showed no significant difference between EcO and control (log₂FC = -2.12, adjusted *P* = 0.58).

Smooth muscle genes exhibited the expected enrichment in ovarian lesions. MYH11, ACTA2, DES, and TAGLN were all significantly upregulated in EcO versus control (log₂FC range: 2.68–3.87, all adjusted *P* < 0.01), and showed consistently higher expression in EcO than EcP in the direct comparison (all log₂FC < 0, Fig. 2C), consistent with the fibromuscular phenotype of ovarian endometriomas. Immune checkpoint molecules CTLA4 and PDCD1 were significantly upregulated in EcP versus control (log₂FC = 2.30 and 1.62, respectively; both adjusted *P* < 0.05, Fig. 2C), supporting the ‘immune-hot’ characterization of peritoneal lesions. NNMT expression did not reach statistical significance in patient-level analysis, likely due to the limited sample size of the EcO group (*n* = 4) and its restricted expression in specific epithelial subsets. The differential expression of key genes across tissue types was further visualized using dot plots (Fig. 2D), confirming the selective enrichment of CCL19 and CXCL13 in EcP, and the downregulation of ESR1 and PGR in both lesion types.

Receiver operating characteristic analysis further validated the diagnostic potential of hormone receptor downregulation, with *ESR1* (AUC = 0.666) and *PGR* (AUC = 0.633) indicating a moderate ability to distinguish endometriotic lesions from control tissues (*ESR1*: AUC = 0.666; *PGR*: AUC = 0.633; Supplementary Fig. 3C), supporting the biological relevance of the transcriptional changes identified in our study.

These pseudo-bulk validation results confirm that the divergent immunological and stromal programs identified in this study reflect genuine biological differences between endometriosis subtypes rather than technical artifacts or cell-level pseudoreplication.

### Characterization of *CCL19* + inflammatory stromal cells and key molecular signatures

**Fig. 3:**
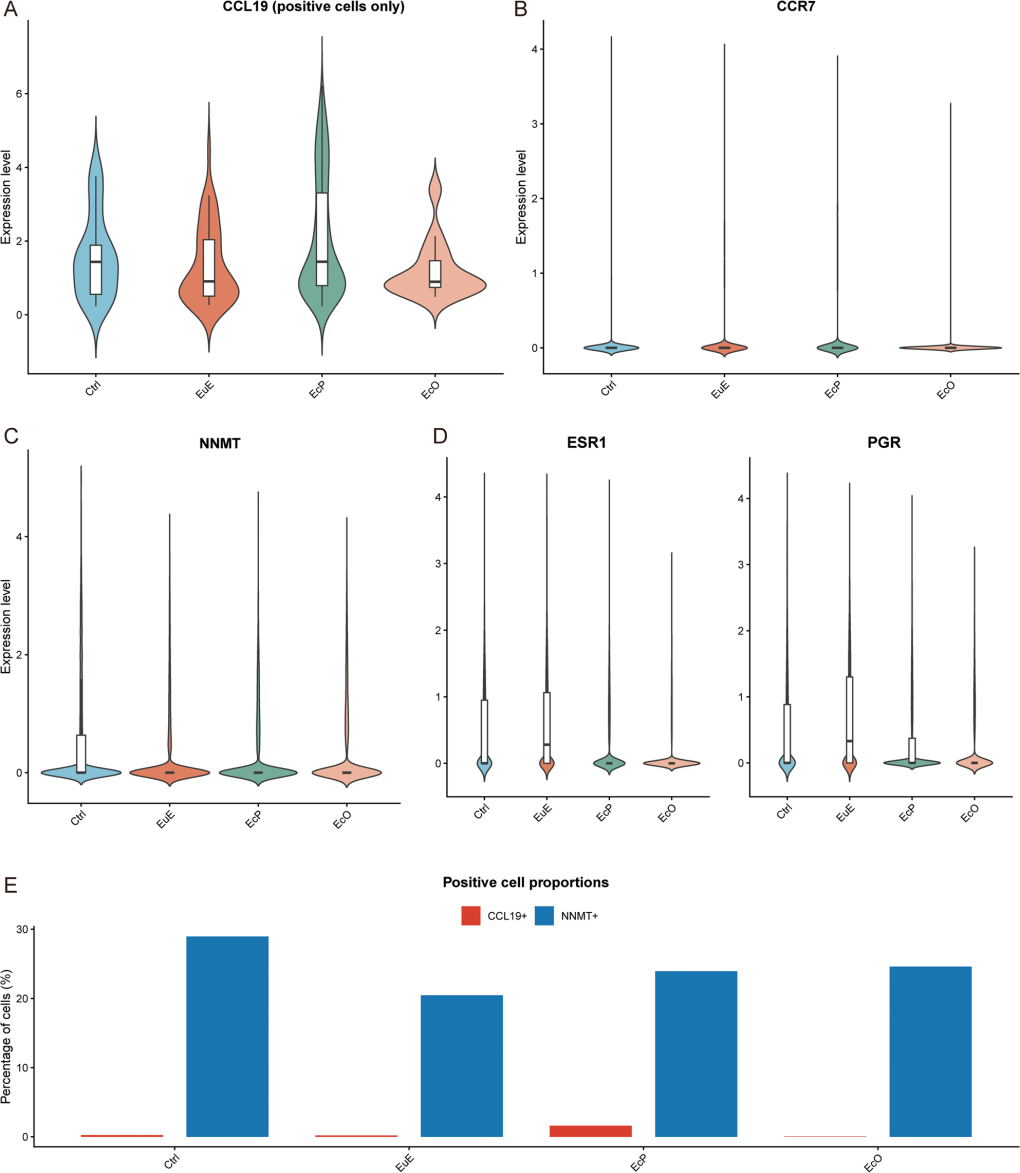
Key molecular signatures distinguishing EcP and EcO. **A** Violin plots showing *CCL19* expression distribution across Ctrl, EuE, EcP, and EcO. Individual points represent *CCL19*-positive cells (expression > 0). *CCL19* expression is highest in EcP (Wilcoxon test, *P* < 0.001 vs. Ctrl and EcO). **B** Violin plots showing *CCR7* expression distribution across the four tissue groups. *CCR7* is specifically enriched in EcP (*P* < 0.001 vs. Ctrl). **C** Violin plots showing *NNMT* expression distribution. *NNMT* expression is highest in EcO (EcO vs. Ctrl: *P* < 0.001; EcO vs. EcP: *P* < 0.001). **D** Violin plots showing hormone receptor expression. Both *ESR1* (left) and *PGR* (right) are significantly downregulated in endometriotic lesions compared to Ctrl (*P* < 0.001 for EcP and EcO vs. Ctrl). **E** Bar plot showing the proportion of *CCL19*-positive and *NNMT*-positive cells in each tissue group. *CCL19*-positive cells are most abundant in EcP (1.61%, 374/23,251), representing a 6.4-fold enrichment over Ctrl (0.25%, 26/10,351, proportion test, *P* < 0.001). *NNMT*-positive cells show comparable proportions across groups, with 24.6% in EcO (5,813/23,634), 29.0% in Ctrl, 23.9% in EcP, and 20.5% in EuE

To complement these patient-level findings with cellular-level resolution, we next examined the expression patterns of key molecular signatures using single-cell data. The analysis of *CCL19* expression revealed distinct tissue-specific patterns (Fig. 3). Violin plots illustrate *CCL19* expression distribution across tissue groups, with individual points representing *CCL19*-positive cells (expression > 0). *CCL19* expression was the highest in EcP, with a 7.6-fold increase over the Ctrl within positive cells (Wilcoxon test, *P* < 0.001) (Fig. 3A). *CCR7*, the receptor for *CCL19*, showed similar enrichment in EcP, with expression levels 2.3-fold higher than those in controls (*P* < 0.001) (Fig. 3B). In contrast, EcO exhibited the highest expression of *NNMT*, an enzyme implicated in cell survival (EcO vs. Ctrl: *P* < 0.001; EcO vs. EcP: *P* < 0.001) (Fig. 3C). Both estrogen receptor alpha (*ESR1*) and progesterone receptor (*PGR*) were markedly downregulated in endometriotic lesions relative to the Ctrl (Fig. 3D). *ESR1* expression was reduced to 51% of the control levels in EcP (*P* < 0.001) and only 17% in EcO (*P* < 0.001). Quantification of positive cell proportions revealed that EcP contained the highest proportion of *CCL19*-expressing cells (374/23,251, 1.61%), representing a 6.4-fold enrichment compared with the Ctrl (26/10,351, 0.25%, proportion test, *P* < 0.001). In contrast, EcO showed minimal *CCL19* expression (14/23,634, 0.06%, *P* < 0.001 vs. EcP). For *NNMT*, the proportion of positive cells was comparable across tissue types, with 24.6% in EcO (5,813/23,634), 29.0% in control (2,997/10,351), 23.9% in EcP (5,564/23,251), and 20.5% in EuE (5,003/24,440) (Fig. 3E). Per-patient analysis confirmed that CCL19 + cells were consistently enriched in EcP across all eight patients (range: 0.06%–2.99%, mean 1.44% ± 0.96%), with only one patient (E07) showing a relatively low frequency (0.06%). In contrast, EcO patients showed uniformly low frequencies (range: 0%–0.16%, mean 0.06% ± 0.08%), and control patients showed similarly low levels (range: 0%–0.33%, mean 0.19% ± 0.17%) (Supplementary Fig. 5A). These findings confirmed *CCL19* is predominantly expressed in EcP, while *NNMT* shows broader expression with modest enrichment in ovarian lesions, highlighting the divergent molecular programs of the two endometriosis subtypes.

To further characterize the immune microenvironment in endometriosis lesions, we performed detailed immune cell subpopulation analysis (Supplementary Fig. 5). UMAP visualization of immune cells identified across all tissue types revealed distinct clustering patterns, with immune cells from EcP forming separate clusters from those in the control and EuE groups, suggesting lesion-specific immune phenotypes (Supplementary Fig. 5B). Expression analysis of lineage-specific markers confirmed the presence of diverse immune cell subsets, with distinct expression patterns across tissue types (Supplementary Fig. 5C). T cell markers (*CD3D*, *CD3E*) showed the highest expression in EcP (mean expression 0.41–0.43, expressed in 19–23% of cells), while EcO exhibited the lowest levels (mean 0.15, 7–8% of cells). CD4 + T cell markers (*CD4*, *IL2RA*) were also most abundant in EcP, with *CD4* expressed in 17.2% of EcP cells compared to 7.1% in EcO. CD8 + T cell markers (*CD8A*, *CD8B*) showed similar enrichment in EcP. B cell markers (*CD79A*, *MS4A1*) were predominantly found in Ctrl and EuE, with minimal expression in lesions. Macrophage markers (*CD68*, *CD163*) were most highly expressed in EcP (*CD68*: mean 0.29, 24.5% of cells; *CD163*: mean 0.16, 10.6% of cells), consistent with the macrophage-rich phenotype of peritoneal lesions. NK cell markers (*NKG7*, *GNLY*) showed the highest expression in Ctrl and EcP, with lower levels in EcO.

The distribution of major immune subsets further illustrated the enrichment of macrophages and T cells in EcP, while EcO showed relative immune sparsity (Supplementary Fig. 5D). Differential expression analysis within immune cells highlighted key genes associated with immune activation and regulation (Supplementary Fig. 5E). Pathway-level analysis confirmed elevated immune checkpoint and chemokine signaling in EcP immune cells (Supplementary Fig. 5F-G). To identify cellular populations that may respond to CCL19 signaling, we examined CCR7 expression across cell types at single-cell resolution (Supplementary Fig. 5H). CCR7 was predominantly expressed in immune cells, with the highest proportions in B cells (43.6%) and T cells (16.6%), whereas stromal cells showed minimal expression (1.16%) (Supplementary Fig. 5H). Combined with our finding that CCL19 is primarily produced by stromal cells (1.61%), these data support a paracrine signaling model in which CCL19-expressing stromal cells recruit CCR7-expressing immune cells within the peritoneal lesion microenvironment (Supplementary Fig. 5H). Extended therapeutic target expression patterns across tissue types are provided in Supplementary Fig. 5I.

### Stromal and vascular cell heterogeneity

**Fig. 4:**
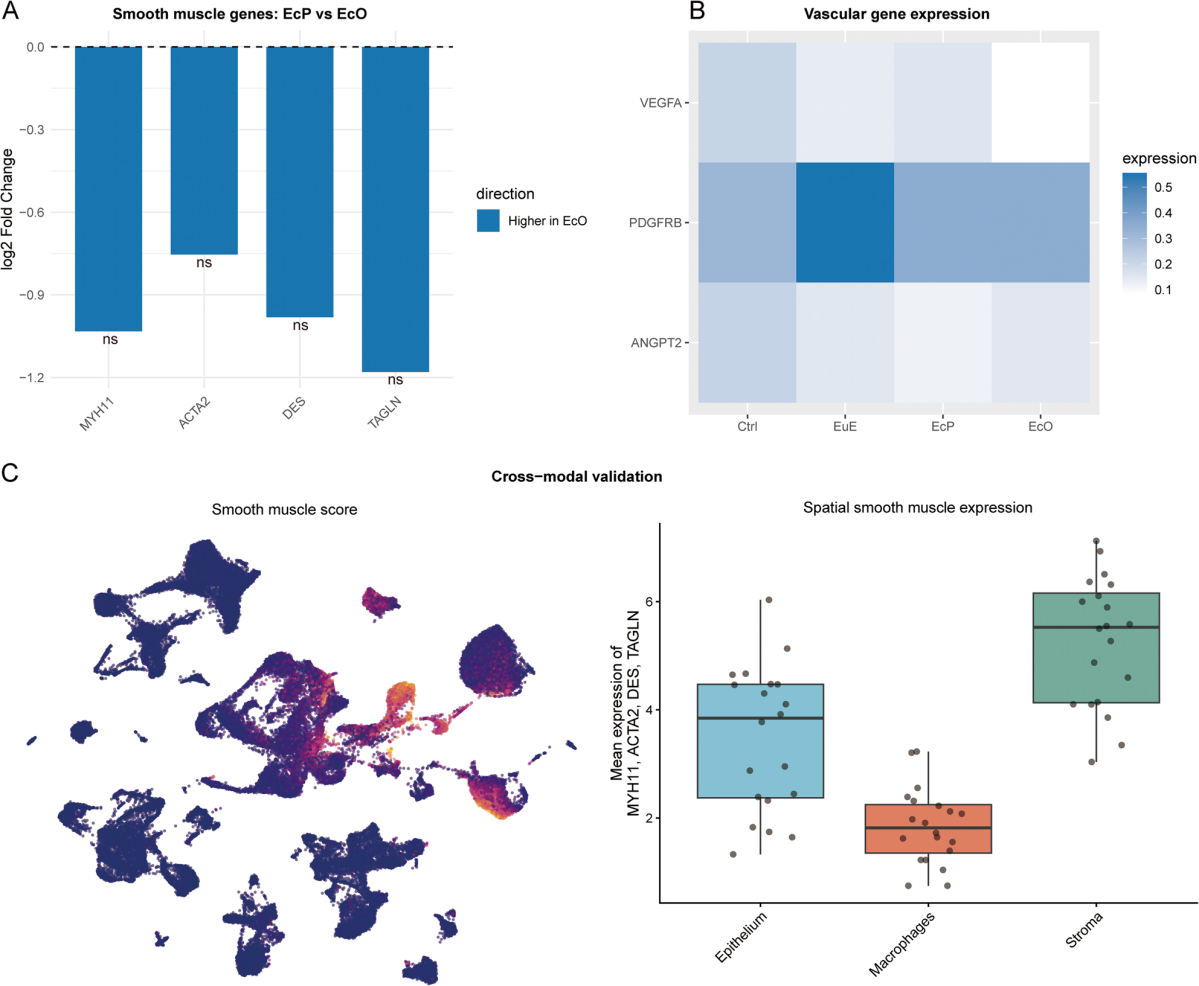
Stromal and vascular cell heterogeneity in endometriosis subtypes. **A** Bar plot showing smooth muscle gene expression from patient-level pseudo-bulk analysis comparing EcP and EcO. All four smooth muscle markers (*MYH11*, *ACTA2*, *DES*, *TAGLN*) show consistently higher expression in EcO (negative log₂FC), although differences did not reach statistical significance at the patient level (adjusted *P* > 0.05, ns = not significant). **B** Heatmap showing mean expression of vascular genes (*VEGFA*, *PDGFRB*, *ANGPT2*) across tissue types (Ctrl, EuE, EcP, EcO). Color intensity represents average expression level. **C** Cross-modal validation of smooth muscle phenotype. Left: UMAP visualization of single cells colored by smooth muscle score (average expression of *MYH11*, *ACTA2*, *DES*, and *TAGLN*). EcO cells exhibit the highest smooth muscle scores. Right: Spatial transcriptomic analysis showing smooth muscle gene expression across cellular compartments (Macrophages, Epithelium, Stroma) in peritoneal lesions. Box plots indicate median and interquartile range; points represent individual spatial segments

Analysis of stromal and vascular cell heterogeneity revealed distinct expression patterns between EcP and EcO (Fig. 4). Using patient-level pseudo-bulk analysis, we examined the expression of key smooth muscle genes. Although the direct comparison between EcP and EcO did not reach statistical significance for individual genes (all adjusted *P* > 0.05), all four smooth muscle markers—*MYH11*, *ACTA2*, *DES*, and *TAGLN*—showed consistently higher expression in EcO compared to EcP (log₂FC range: -1.18 to -0.75), indicating a trend toward enhanced smooth muscle differentiation in ovarian lesions (Fig. 4A). Vascular gene expression analysis revealed distinct patterns across tissue types (Fig. 4B). *VEGFA* showed the highest expression in Ctrl, while *PDGFRB* was maximally expressed in EuE. Notably, *ANGPT2* expression was elevated in EcO compared to other groups, suggesting differential regulation of vascular stabilization pathways in EcO. Integrated cross-modal analysis confirmed the smooth muscle phenotype of EcO (Fig. 4C). UMAP visualization of smooth muscle scores (based on *MYH11*, *ACTA2*, *DES*, and *TAGLN*) across 81,676 single cells revealed that EcO exhibited the most pronounced smooth muscle signature (Fig. 4C, left). Spatial transcriptomic analysis further demonstrated that smooth muscle gene expression was predominantly localized to the stromal compartment, with the highest expression observed in stromal segments of peritoneal lesions (Fig. 4C, right). This multimodal approach provides complementary evidence of the enhanced smooth muscle differentiation characteristics of EcO.

### Spatial compartmentalization and intercellular communication networks

**Fig. 5:**
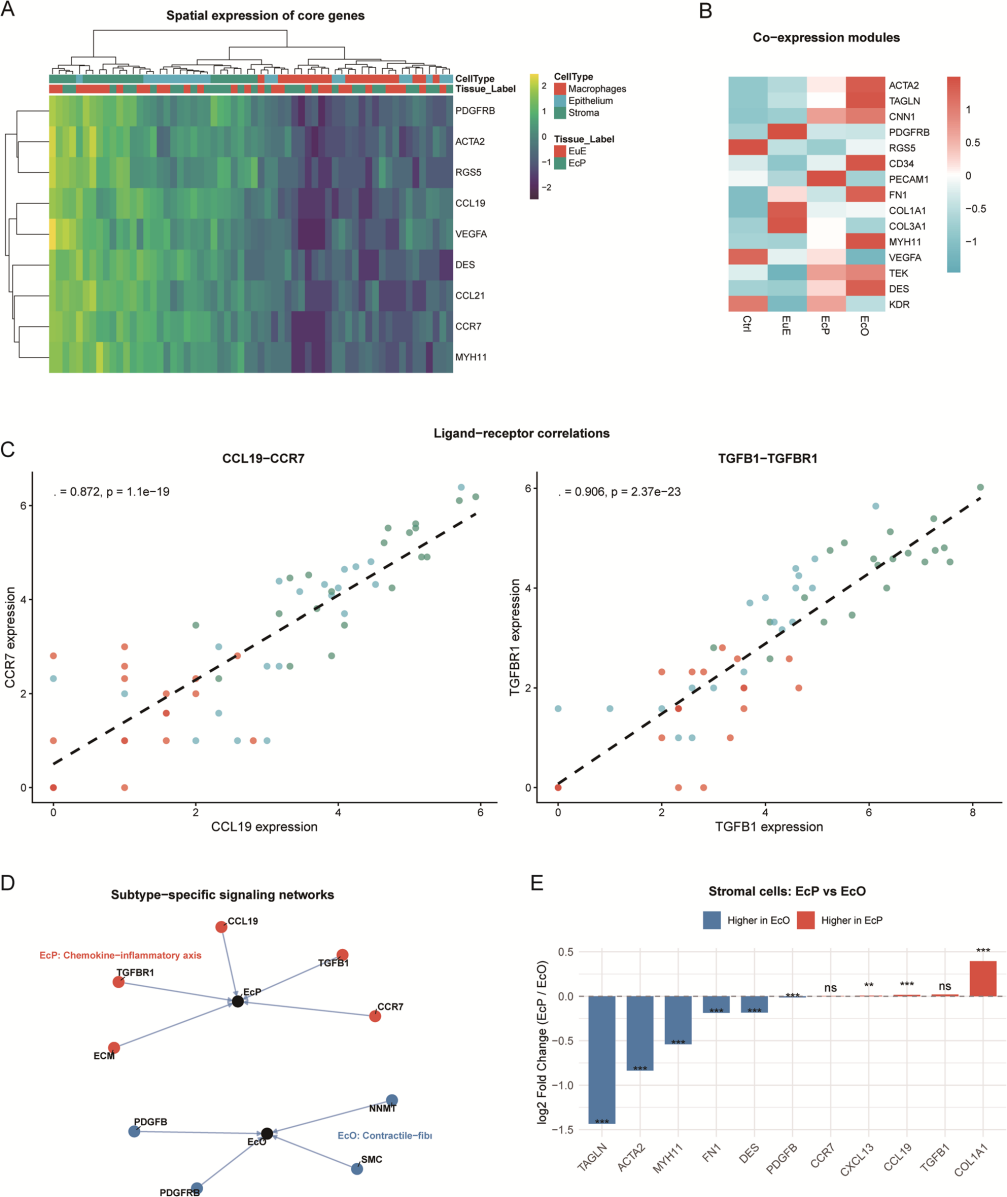
Spatial organization and intercellular communication networks in endometriosis subtypes. **A** Spatial expression heatmap of core genes across cellular compartments (Macrophages, Epithelium, Stroma) in EuE and EcP. Color intensity represents row-scaled expression levels. **B** Co-expression module heatmap showing average expression of 15 vascular, smooth muscle, and ECM genes across tissue types (Ctrl, EuE, EcP, EcO). Genes are ordered by functional categories. **C** Ligand-receptor correlation plots from spatial transcriptomics data. Left: CCL19 vs. CCR7 (Spearman ρ = 0.872, *P* = 1.1 × 10⁻¹⁹). Right: TGFB1 vs. TGFBR1 (Spearman ρ = 0.906, *P* = 2.4 × 10⁻²³). Points are colored by cellular compartment. **D** Schematic model of subtype-specific signaling networks. EcP are characterized by a chemokine-inflammatory axis involving CCL19-CCR7 signaling, which may activate downstream TGF-β pathways and ECM remodeling. EcO exhibit a contractile-fibrotic program dominated by PDGF signaling and smooth muscle differentiation. **E** Bar plot showing differential expression of key genes in stromal cells between EcP and EcO. All genes shown are significantly differentially expressed (adjusted *P* < 0.05) except where indicated. Positive log₂FC indicates higher expression in EcP stromal cells; negative log₂FC indicates higher expression in EcO stromal cells. Significance: *P < 0.05, **P < 0.01, ***P < 0.001; ns, not significant

Spatial transcriptomic analysis revealed highly organized distribution of pathogenic signatures in peritoneal lesions (Fig. 5A). Smooth muscle markers (*ACTA2*, *MYH11*, *DES*), pericyte gene *RGS5*, and chemokine axis genes (*CCL19*,* CCR7*,* CCL21*) was predominantly localized to stromal compartments, where *CCL19* and *CCR7* expression showed strong correlation (ρ = 0.872, *P* = 1.1 × 10⁻^19^), suggesting an active, stroma-centric chemotactic signaling hub. Angiogenesis-related genes (*VEGFA* and *PDGFRB*) exhibited broader distribution across all compartments.

Unsupervised hierarchical clustering of 15 vascular, smooth muscle, and ECM genes revealed distinct transcriptional modules across tissue types (Fig. 5B). Smooth muscle genes (*ACTA2*, *TAGLN*, *MYH11*, *DES*) showed progressive enrichment from Ctrl to EcO, while ECM genes (*COL1A1*, *COL3A1*, *FN1*) were most abundant in EuE.

Ligand-receptor analysis demonstrated strong correlations for CCL19-CCR7 (ρ = 0.872) and TGFB1-TGFBR1 (ρ = 0.906, *P* = 2.4 × 10⁻^23^) in peritoneal lesions (Fig. 5C). Based on these findings, we propose distinct signaling networks for the two subtypes (Fig. 5D). Peritoneal lesions are characterized by a chemokine-inflammatory axis involving *CCL19*-*CCR7* signaling, which may activate downstream TGF-β pathways and subsequent extracellular matrix remodeling. In contrast, ovarian lesions exhibit a contractile-fibrotic program dominated by PDGF signaling and smooth muscle differentiation, consistent with their fibromuscular phenotype. To determine whether these differences reflect cell-intrinsic reprogramming rather than compositional shifts, we compared gene expression within stromal cells, the primary source of chemokine and ECM signals. Stromal cells in EcP showed significant upregulation of *CCL19* (log₂FC = 0.016, adjusted *P* < 0.001) and *COL1A1* (log₂FC = 0.39, adjusted *P* < 0.001), while those in EcO exhibited enrichment of smooth muscle-associated genes including *TAGLN* (log₂FC = -1.43, adjusted *P* < 0.001) and *ACTA2* (log₂FC = -0.84, adjusted *P* < 0.001) (Fig. 5E). These results revealed that the divergent programs are driven by cell-intrinsic changes within the stromal lineage.

Extended co-expression and communication analyses are provided in Supplementary Fig. 6. Full GO enrichment results for the three co-expression modules are shown in Supplementary Fig. 6A, complete ligand-receptor heatmaps in Supplementary Fig. 6B, and multiscale comparisons of molecular architectures between subtypes in Supplementary Fig. 6C. To exclude the possibility that the strong CCL19-CCR7 correlation observed in stromal segments was driven by immune cell contamination, we performed composition-adjusted correlation analysis. After controlling for immune cell content (based on expression of CD68, CD3D, CD3E, PTPRC, CD4, and CD8A), the partial correlation between CCL19 and CCR7 remained significant (ρ_partial = 0.708, *P* = 0.033), confirming that the spatial association reflects genuine co-expression patterns rather than technical admixture (Supplementary Fig. 6D).

### Comprehensive immune profiling reveals divergent immunological landscapes

**Fig. 6:**
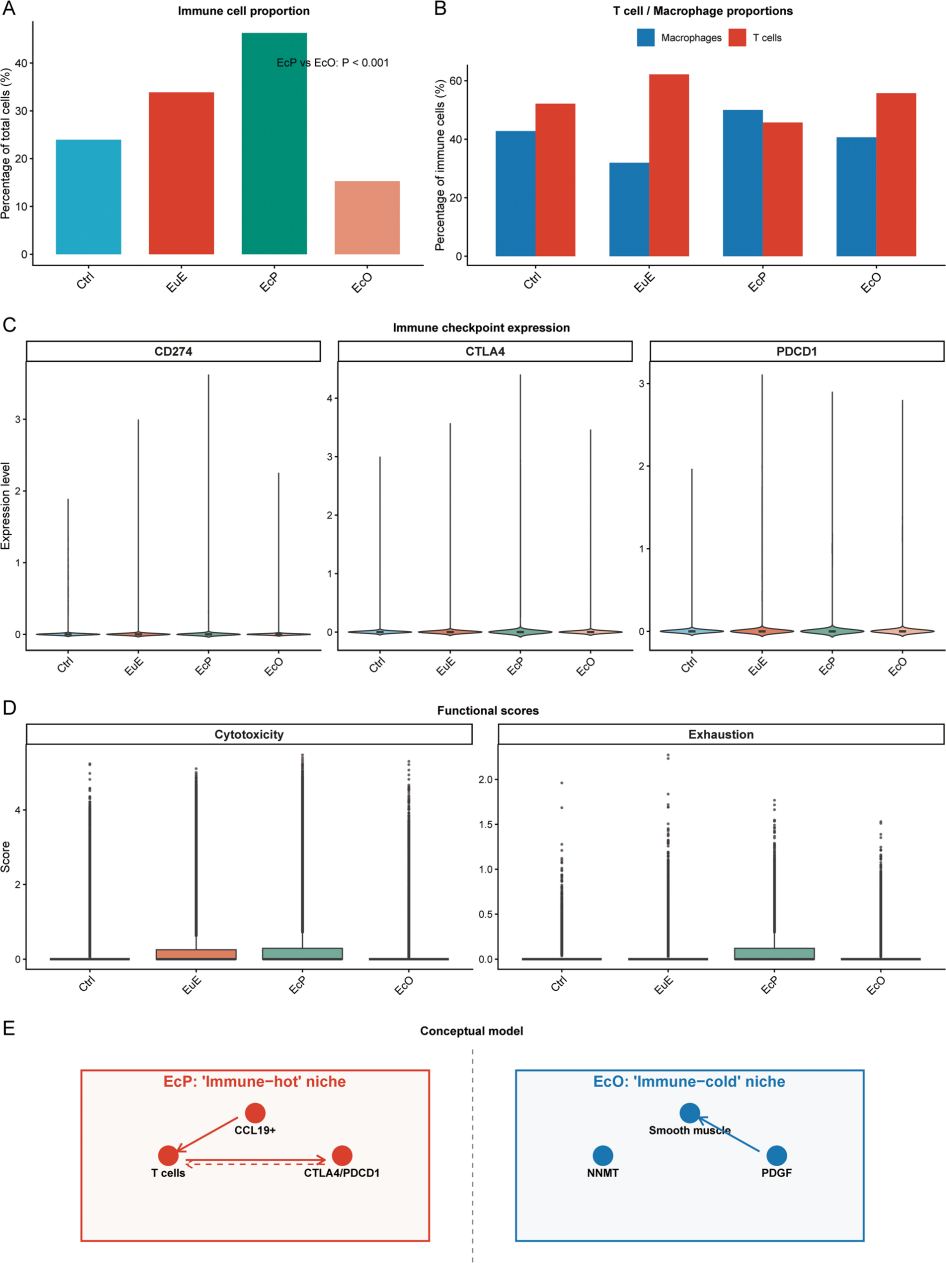
Comprehensive immune profiling reveals divergent immunological landscapes in endometriosis subtypes. **A** Bar plot showing the proportion of immune cells (T cells, macrophages, B cells, and NK cells) across tissue types (Ctrl, EuE, EcP, EcO). EcP contains the highest proportion of immune cells (46.3%), while EcO shows the lowest (15.3%, *P* < 0.001 vs. EcP). **B** Bar plot showing the proportions of T cells and macrophages within the immune cell compartment across tissue types. EcP exhibits a balanced distribution of macrophages (50.0%) and T cells (45.7%), whereas EcO is T cell-rich (55.7%) with fewer macrophages (40.7%). **C** Violin plots showing expression levels of immune checkpoint molecules (CTLA4, PDCD1, CD274) across tissue types. All three checkpoints are significantly upregulated in EcP compared to both Ctrl and EcO (*p* < 0.001 for all comparisons). **D** Box plots showing cytotoxicity and exhaustion scores across tissue types. Cytotoxicity scores (based on GZMB, PRF1, GNLY) are highest in EcP (mean 0.424) and lowest in EcO (mean 0.098, *P* < 0.001). Exhaustion scores (based on CTLA4, PDCD1, LAG3, HAVCR2, TIGIT) are also elevated in EcP (mean 0.083) compared to EcO (mean 0.046, *P* < 0.001). **E** Conceptual model summarizing the divergent immune microenvironments. EcP is characterized as an “immune-hot” niche with relative immune enrichment, featuring abundant CCL19 + perivascular cells, T cells and macrophages, upregulated immune checkpoint molecules, and elevated cytotoxicity. In contrast, EcO represents an “immune-cold” niche with relative immune sparsity, dominated by smooth muscle differentiation, NNMT-mediated epithelial survival, and sparse immune infiltration

Integrated analysis of immune cells revealed striking differences between endometriosis subtypes (Fig. 6). EcP lesions contained the highest proportion of immune cells (46.3% of total cells), followed by EuE (33.9%) and Ctrl (23.9%), while EcO showed the lowest immune infiltration (15.3%, *P* < 0.001 vs. EcP) (Fig. 6A). Among immune cells, the composition of T cells and macrophages varied significantly across tissue types (Fig. 6B). EcP was characterized by a balanced distribution of macrophages (50.0%) and T cells (45.7%), whereas EcO showed a predominance of T cells (55.7%) over macrophages (40.7%). Ctrl and EuE exhibited T cell-rich profiles (52.1% and 62.2%, respectively) with lower macrophage proportions. Immune checkpoint molecules were differentially expressed across groups (Fig. 6C). CTLA4, PDCD1, and CD274 were all significantly upregulated in EcP compared to both Ctrl and EcO (all *P* < 0.001). The highest expression levels were observed in EcP for all three checkpoints, consistent with an “immune-hot” phenotype. Functional scoring revealed enhanced cytotoxicity in EcP immune cells (mean score 0.424) compared to EcO (0.098, *P* < 0.001), while exhaustion scores (based on *CTLA4*, *PDCD1*, *LAG3*, *HAVCR2*, *TIGIT*) were also elevated in EcP (0.083 vs. 0.046 in EcO, *P* < 0.001) (Fig. 6D). These findings collectively support a model where EcP represents an “immune-hot” niche with relative immune enrichment, characterized by active immune responses and checkpoint-mediated regulation, while EcO constitutes an “immune-cold” niche with relative immune sparsity, characterized by sparse immune infiltration and reduced immune activity (Fig. 6E).

### Therapeutic target expression analysis

**Fig. 7:**
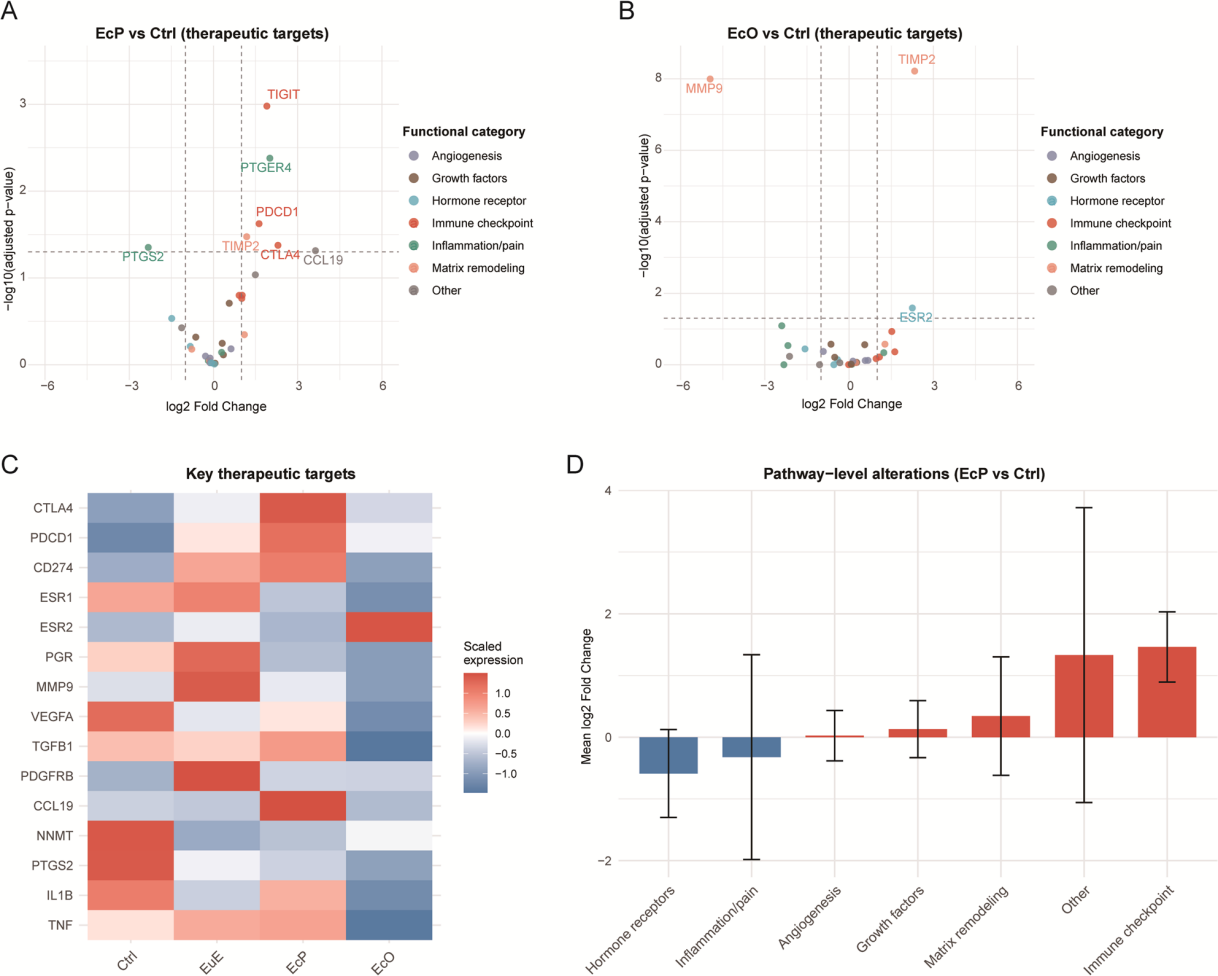
Therapeutic target expression analysis in endometriosis subtypes. **A** Volcano plot of differentially expressed therapeutic target genes in EcP vs. Ctrl. Points are colored by functional category. Key significantly altered genes are labeled. Dashed lines: significance thresholds (|log₂FC| > 0.5, adjusted *P* < 0.05). **B** Volcano plot for EcO vs. Ctrl, following the same visualization scheme. **C** Heatmap showing row-scaled expression of 15 key therapeutic target genes across tissue types (Ctrl, EuE, EcP, EcO). Genes are grouped by functional category. **D** Pathway-level alterations: mean log₂ fold change (vs. Ctrl) for each target category in EcP (red). Error bars represent SD. Numbers indicate gene counts per category

We systematically analyzed the expression of 31 clinically relevant therapeutic target genes across endometriosis subtypes. Differential expression analysis revealed distinct subtype-specific patterns (Fig. 7A-B). In peritoneal lesions (EcP vs. Ctrl), immune checkpoint molecules showed the most prominent upregulation: *CTLA4* (log₂FC = 2.30, adjusted *P* = 0.042), *PDCD1* (log₂FC = 1.62, adjusted *P* = 0.024), and *TIGIT* (log₂FC = 1.90, adjusted *P* = 0.001), and the prostaglandin receptor *PTGER4* (log₂FC = 2.01, adjusted *P* = 0.004). CCL19 was also significantly upregulated (log₂FC = 3.63, adjusted *P* = 0.049), whereas COX-2 (*PTGS2*) was downregulated (log₂FC = -2.32, adjusted *P* = 0.045) (Fig. 7A).

In ovarian lesions (EcO vs. Ctrl), a distinct pattern emerged. Matrix metalloproteinase 9 (*MMP9*) exhibited dramatic downregulation (log₂FC = -4.95, adjusted *P* < 0.001), while TIMP2 was significantly upregulated (log₂FC = 2.34, adjusted *P* < 0.001). Estrogen receptor beta 2 (ESR2) showed modest upregulation (log₂FC = 2.25, adjusted *P* = 0.026), contrasting with the downregulation of ESR1 and PGR observed in both lesion types (Fig. 7B).

Heatmap analysis of 15 key therapeutic targets revealed clear clustering by tissue type (Fig. 7C). Immune checkpoint genes (*CTLA4*, *PDCD1* and *CD274*) were specifically enriched in EcP, while hormone receptor genes (*ESR1* and *PGR*) showed highest expression in Ctrl and EuE. Notably, *NNMT* and *ESR2* exhibited elevated expression in EcO, consistent with their roles in ovarian lesion biology.

Pathway-level quantification confirmed that immunomodulatory targets had the highest mean upregulation in EcP (mean log₂FC = 1.46), whereas hormone receptors showed the greatest downregulation in EcO (mean log₂FC = -0.59). Matrix remodeling and growth factor pathways exhibited modest positive changes, while inflammation/pain targets showed variable patterns (Fig. 7D).

These findings highlight subtype-specific therapeutic vulnerabilities: immunomodulatory strategies targeting immune checkpoints or the CCL19-CCR7 axis may be particularly relevant for EcP, while EcO may be more amenable to stroma-targeted approaches such as NNMT inhibition or anti-fibrotic agents.

### Quality control of spatial transcriptomics data

Quality control analysis confirmed the robustness of the spatial transcriptomic data. Total RNA counts and gene detection were consistent across all 60 spatial segments, with median counts of 196,140 per segment and a median of 17,924 genes detected per segment (Supplementary Fig. 1C). A positive correlation was observed between total RNA counts and detected genes (Pearson *r* = 0.575, *P* = 1.58 × 10⁻⁶), indicating technical reliability (Supplementary Fig. 1D). Importantly, the Q3 normalization method used for these data does not rely on housekeeping genes, ensuring that the observed differences across compartments are not confounded by variations in housekeeping gene expression.

To assess potential batch effects, we performed PCA on the spatial data. Biological factors (tissue type and compartment) explained 60.9% of the total variance, while technical batch effects accounted for only 12.3% (Supplementary Fig. 1E), indicating that the observed transcriptional variation reflects primarily biological differences rather than technical artifacts. Detailed quality metrics are provided in Supplementary Fig. 1.

## Discussion

Our integrated analysis revealed that EcP and EcO were maintained by fundamentally distinct molecular programs. EcP is characterized by specific enrichment of the *CCL19*/*CCR7* chemokine axis, with *CCL19* expression within positive cells 7.6-fold higher than that in the Ctrl and *CCR7* showing a 2.3-fold increase. In contrast, EcO exhibited marked upregulation of *NNMT* along with pronounced smooth muscle differentiation. These molecular signatures, combined with differential hormone receptor expression and immune checkpoint profiles, indicate that lesion location dictates unique pathogenic mechanisms, with direct implications for subtype-specific therapeutic strategies.

The integrative transcriptomic approach employed in this study extends previous endometriosis research by directly linking single-cell transcriptional profiles with spatial organization. While earlier studies have characterized either cellular composition or spatial architecture in isolation, our cross-modal framework reveals how the distinct immunological and stromal programs of EcP and EcO are compartmentalized within the tissue microenvironment [11, 14].

### Divergent immunological landscapes: an immune-hot chemokine niche in EcP versus an immune-cold fibromuscular niche in EcO

The most striking divergence between lesion types lies in core stromal-immune ecosystems. EcP constructs a chemokine-rich, immune-active niche centered on the *CCL19*-*CCR7* axis, with *CCL19*-positive cells comprising 1.61% of the cellular population, a six-fold increase over the Ctrl. This finding resonates with functional studies demonstrating that the *CCL19*/*CCR7* axis activates PI3K/Akt signaling to promote endometrial stromal cell proliferation and invasion [16]. Importantly, this chemokine-driven microenvironment aligns with established knowledge regarding fibrosis in EcP, where active extracellular matrix remodeling and stromal activation create a dynamic, vascularized fibrotic stroma [32, 33]. Thus, *CCL19* in EcP likely serves a dual function: recruiting immune cells to create an inflammatory milieu while simultaneously driving local lesion expansion through autocrine/paracrine signaling. This self-reinforcing “inflammatory engine” may explain the aggressive, infiltrative nature of peritoneal disease and its strong association with pelvic pain, as previously suggested by clinical observations [34].

In stark contrast, EcO eschews this chemokine-driven inflammation for structural encapsulation and cellular survival. Rather than constructing an immune-recruiting niche, EcO invests in fibromuscular differentiation, a phenomenon well documented in EcO [35]. Our data revealed pronounced smooth muscle differentiation in EcO, consistent with their fibromuscular characteristics that provide structural support to cyst walls [35]. Concurrently, EcO exhibits the highest expression of NNMT, a key enzyme recently implicated in conferring resistance to apoptosis in the endometriotic epithelium [17]. This survival advantage, combined with their structural differentiation, enables EcO to form well-demarcated, persistent cysts, essentially building a “fibrotic fortress” that prioritizes structural integrity over inflammatory signaling. This strategy may explain why EcO often presents as isolated cystic masses rather than as diffuse peritoneal disease.

Our comprehensive immune profiling revealed that EcP lesions contain nearly three-fold more immune cells than EcO (46.3% vs. 15.3%), with distinct cellular compositions: EcP is enriched for macrophages and T cells, whereas EcO shows minimal immune infiltration. Furthermore, immune checkpoint molecules CTLA4, PDCD1, and CD274 are significantly upregulated in EcP, suggesting an adaptive immune regulatory state. Pathway analysis demonstrated that while checkpoint expression is highest in EcO, the CCL19-CCR7 chemokine axis and cytotoxicity programs are specifically elevated in EcP.

Collectively, our data paint a picture of two fundamentally distinct immunological landscapes. The EcP microenvironment, rich in *CCL19* + perivascular cells, T cells, and macrophages with upregulated immune checkpoint molecules (CTLA4, PDCD1), exhibits relative immune enrichment, resembling an ‘immune-hot’ or inflamed tumor microenvironment [21–23]. This raises the possibility that EcP lesions could be investigated for susceptibility to immunomodulatory therapies, though this hypothesis requires direct experimental testing. In contrast, the EcO niche, dominated by smooth muscle cells and fibroblasts with minimal leukocyte infiltration and low chemokine expression, shows relative immune sparsity, aligns with an ‘immune-cold, fibrotic’ or immunologically deserted phenotype, potentially explaining its resistance to conventional anti-inflammatory treatments and highlighting the need for alternative strategies targeting stromal resilience. This “immune-hot” versus “immune-cold” dichotomy extends beyond mere cellular abundance to functional states: EcP immune cells exhibit elevated cytotoxicity and chemokine signaling, whereas EcO shows predominant interferon responses and checkpoint expression even within its sparse immune compartment. These functional differences may dictate differential responses to immunotherapies. The high variance in immune response scores observed in EcP suggests that peritoneal lesions contain a mixture of immune-active and immune-quiescent cells, reflecting the complex and heterogeneous nature of the lesion microenvironment. In contrast, the elevated TGF-β signaling points to a more consistently activated pathway that may play a key role in fibrosis and immune regulation in EcP.

It is important to note that the near-perfect spatial correlation between *CCL19* and *CCR7* (ρ = 0.872), while striking, reflects tight co-regulation rather than direct evidence of causation. This strong collinearity may arise from the spatial co-localization of *CCL19*-producing stromal cells and *CCR7*-expressing immune cells within the same niche, rather than from a direct ligand-receptor interaction driving both genes’ expression. To further investigate this, we performed single-cell resolution analysis, which revealed that CCL19 is predominantly expressed in stromal cells (1.61%), while CCR7 is highly enriched in B cells (43.6%) and T cells (16.6%)—a cellular distribution pattern consistent with a paracrine signaling model. Moreover, after controlling for potential immune cell contamination in spatial segments, the partial correlation between CCL19 and CCR7 remained significant (ρ_partial = 0.708, *P* = 0.033), supporting that the observed spatial association reflects genuine co-expression patterns rather than technical admixture. Thus, while our data support a functional chemokine signaling hub in peritoneal lesions, they cannot distinguish whether *CCL19* and *CCR7* independently contribute to the phenotype or if one is primary and the other a consequence of shared regulatory programs. Functional studies, such as *CCL19*/*CCR7* blockade experiments, will be essential to dissect their respective roles.

### Epithelial reprogramming: from uniform origin to divergent roles

The endometrial epithelial cell, the presumed cell of origin in endometriosis, undergoes remarkable phenotypic specialization depending on its final anatomical destination. In EcP, epithelial cells adopt a pro-inflammatory macrophage-educating phenotype, expressing high levels of complement components and chemokines that recruit and activate myeloid cells [14]. This aligns with spatial transcriptomic evidence that epithelium-macrophage crosstalk is central to EcP biology and supports the concept that epithelial cells are active participants in creating disease-permissive microenvironments [14]. Thus, the peritoneal epithelium functions as an inflammatory orchestrator, actively shaping its microenvironment, which is consistent with observations of altered epithelial characteristics in endometriosis [34].

Conversely, the EcO epithelium has a different survival strategy. Beyond NNMT-mediated apoptosis resistance, these cells exhibit features reminiscent of a stressed adaptive state. Recent single-cell profiling has identified distinct hormonal and inflammatory signatures in endometriosis-constituting cells, with the ovarian epithelium showing particular dysregulation of estrogen response pathways [18]. This specialized epithelial adaptation likely reflects the unique challenges of the ovarian microenvironment, including the exposure to follicular fluid components, cyclical hormonal fluctuations, and mechanical constraints of the ovarian cortex. Thus, the epithelium in ovarian cysts may be less an inflammatory instigator and a more resilient survivor, fortified against extrinsic stresses, an adaptation that may contribute to the chronic persistence of EcO.

Notably, comparison between EuE and Ctrl revealed distinct molecular features that may reflect intrinsic endometrial abnormalities associated with endometriosis. EuE exhibited intermediate proportions of multiple cell lineages, with a trend toward reduced epithelial and stromal cells compared to Ctrl. At the transcriptional level, EuE showed upregulation of genes involved in inflammatory responses and extracellular matrix remodeling relative to Ctrl, consistent with the concept of a “primed” endometrial phenotype that may predispose to ectopic implantation and lesion establishment. These findings align with previous reports of endometrial dysfunction in endometriosis patients and suggest that EuE carries disease-associated molecular alterations that contribute to the pathophysiology of endometriosis [20].

### Hormonal dysregulation: a common theme with subtype-specific manifestations

Both lesion types exhibited marked dysregulation of steroid hormone signaling but with important distinctions. We confirmed widespread downregulation of estrogen receptor alpha (ESR1) and progesterone receptor (PGR) across endometriotic lesions, a phenomenon previously linked to hormonal resistance and treatment variability [36]. However, the functional consequences of this downregulation may differ according to the lesion type. In EcP, reduced hormone receptor expression may facilitate escape from hormonal regulation while maintaining sensitivity to inflammatory stimuli. In EcO, hormone resistance combines NNMT-mediated survival pathways and extensive fibromuscular differentiation to create a triply resilient phenotype resistant to hormonal therapies, physiological apoptotic signals, and mechanical disruption.

Our analysis also revealed subtype-specific patterns in alternative hormonal pathways. The relative preservation of ESR2 in some EcO suggests compensatory adaptations, whereas differences in GPER1 expression suggest non-classical estrogen signaling variations. These findings align with the emerging understanding of complex hormone signaling in endometriosis [37] and underscore that hormonal dysregulation is not a monolithic defect, but a spectrum of adaptations tuned to local microenvironmental demands.

The mechanisms underlying ESR1 and PGR downregulation in endometriotic lesions remain to be elucidated. Several possibilities could account for this observation. First, epigenetic silencing via DNA methylation or histone modifications has been implicated in hormone receptor regulation in various cancers and could play a role in endometriosis [38]. Second, inflammatory cytokines such as TNF-α and IL-1β, which are elevated in the endometriotic microenvironment, may contribute to ESR1 repression. A recent study in ectopic endometrial tissues reported coordinated dysregulation characterized by upregulated TNF-α expression and concurrent downregulation of ESR1 and PGR, suggesting a potential link between local inflammation and hormone receptor suppression [39]. Third, post-translational degradation via the ubiquitin-proteasome pathway may contribute to reduced receptor protein levels. Our transcriptomic data cannot distinguish between these possibilities, as they reflect steady-state mRNA levels rather than direct measurements of epigenetic status, cytokine signaling, or protein stability. Future studies incorporating epigenetic profiling, cytokine measurements, and protein-level analyses will be essential to dissect the mechanisms driving hormone receptor downregulation and to identify potential therapeutic strategies for restoring hormonal sensitivity in endometriosis lesions.

### Therapeutic implications: from one-size-fits-all to precision targeting

Our comparative analysis challenges the notion that endometriosis is a single disease entity that requires uniform treatment. Instead, we propose a precision medicine framework where therapeutic strategy is guided by lesion molecular subtype, an approach increasingly recognized as necessary for complex gynecological disorders [40, 41].

For EcP, the clear dominance of the *CCL19*-*CCR7* axis suggests promising therapeutic opportunities. Our immune profiling further supports this strategy by demonstrating that EcP lesions are not only enriched for the CCL19-CCR7 axis but also exhibit upregulated immune checkpoint molecules and abundant cytotoxic immune cells. Our therapeutic target analysis further confirms significant upregulation of immune checkpoint genes (*CTLA4*, *PDCD1*, *CD274*) and *CCL19* in EcP. Notably, recent evidence demonstrates that TGF-β1, which is elevated in peritoneal lesions, directly induces PD-1 expression on macrophages via SMAD3/STAT3 cooperative signaling in chronic inflammation, providing a mechanistic link between the chemokine-inflammatory axis and immune checkpoint upregulation observed in EcP [42]. This triple signature—chemokine-driven recruitment, checkpoint-mediated regulation, and cytotoxic potential—suggests that EcP could be a candidate for investigating combined immunomodulatory approaches. Such strategies would target both chemokine signaling and immune checkpoints, though functional studies are needed to establish therapeutic relevance. Small-molecule inhibitors or monoclonal antibodies targeting this axis can simultaneously dampen inflammation and inhibit lesion growth, addressing both inflammatory symptoms and disease progression. The success of similar strategies in autoimmune and inflammatory conditions provides precedent for dual-pathway targeting [37].

EcO, with its distinct biology, demands a different therapeutic logic. Despite its sparse immune infiltration, EcO shows the highest expression of checkpoint molecules in its resident immune cells and strong interferon pathway activation. This suggests that while EcO may be resistant to conventional anti-inflammatory therapies, it might respond to strategies targeting the fibromuscular stroma combined with checkpoint modulation in its limited immune niche. Here, the priority shifts from inflammation suppression to the disruption of structural integrity and induction of apoptosis. Consistent with our findings, *NNMT* showed elevated expression in EcO. NNMT has recently emerged as a key regulator of immunosuppression in the tumor microenvironment. In high-grade serous ovarian cancer, NNMT expression in cancer-associated fibroblasts (CAFs) drives complement secretion and recruitment of myeloid-derived suppressor cells (MDSCs), thereby suppressing CD8 + T cell activation [43]. Notably, a potent and specific NNMT inhibitor developed through high-throughput screening reduced tumor burden and metastasis in multiple mouse cancer models and restored sensitivity to immune checkpoint blockade [43]. Given the shared features between EcO and ovarian cancer microenvironments—particularly the presence of CAFs and an “immune-cold” phenotype—NNMT inhibition represents a promising therapeutic strategy for EcO that warrants investigation in preclinical endometriosis models. These could be combined with antifibrotic agents to disrupt the supportive stromal capsule, targeting the fibromuscular differentiation that characterizes these lesions, and smooth muscle relaxants to alleviate cystic tension and associated pain [35]. For premenopausal women desiring fertility preservation, this targeted approach offers the potential to treat EcO, while minimizing systemic hormonal disruption and preserving ovarian function.

From a clinical classification perspective, our findings support a molecular taxonomy of endometriosis based on immune and stromal features. EcP could be classified as an “immune-hot” subtype potentially amenable to immunotherapy-based approaches, while EcO represents a “stromal-dominant” subtype that may require stroma-targeted therapies. This framework suggests that future clinical trials could stratify patients based on lesion subtype, with EcP patients enrolled in trials testing checkpoint inhibitors or CCL19/CCR7 antagonists, and EcO patients considered for NNMT inhibitors or anti-fibrotic agents. Prospective studies integrating molecular subtyping with treatment outcomes will be essential to validate this precision medicine approach and to develop clinically accessible biomarkers (e.g., immunohistochemistry for CCL19 or NNMT) for subtype identification.

### Limitations and future directions

Several limitations of this study should be considered when interpreting our findings. First, this study is based entirely on publicly available transcriptomic data without experimental validation. While we have added patient-level pseudo-bulk analysis to strengthen statistical rigor, all conclusions regarding cellular programs, signaling pathways, and therapeutic targets remain correlative and hypothesis-generating. Future studies incorporating immunohistochemistry, immunofluorescence, or functional assays (e.g., CCL19/CCR7 blockade, NNMT inhibition) are essential to validate protein expression patterns and establish causality. Second, despite performing pseudo-bulk analysis to account for patient-level biological replication, the sample size remains limited, particularly for the EcO group (*n* = 4 patients). This may have reduced statistical power to detect differences for genes with restricted expression patterns, such as NNMT, which showed consistent trends but did not reach significance at the patient level. Validation in larger independent cohorts is warranted. Third, variance partitioning analysis revealed that while biological factors (tissue type and compartment) explained the majority of transcriptional variance (60.9%) and technical batch effects accounted for a smaller fraction (12.3%), a substantial proportion of variance (26.8%) remained unexplained. This residual variation may reflect several factors, including patient-to-patient heterogeneity within each diagnostic group, unmeasured biological variables such as hormonal status or menstrual cycle phase, and additional technical factors beyond the batch effects assessed. Future studies with larger cohorts and more comprehensive clinical annotation will be needed to dissect these sources of variation and further refine our understanding of the molecular architecture of endometriosis. Fourth, spatial transcriptomic analysis, while providing a crucial tissue context, operates at the segment-level rather than single-cell resolution, and our study focused on established lesions rather than their developmental trajectory. To address the potential confounding effect of mixed cellular composition within spatial segments, we performed composition-adjusted correlation analysis controlling for immune cell content. This analysis confirmed that the strong CCL19-CCR7 correlation persisted after adjustment, supporting the biological relevance of this signaling axis. Future studies employing higher-resolution spatial technologies (e.g., Xenium and MERFISH) could further refine these findings by precisely mapping the cellular neighborhoods of key populations, such as *CCL19* + perivascular or *NNMT*+ epithelial cells. Fifth, a key limitation of our study is the lack of directly matched spatial transcriptomic data for EcO lesions. While our integrative approach allowed us to infer spatial principles by contrasting the well-defined spatial architecture of EcP with single-cell profiles of EcO, future spatial profiling of EcO tissue is essential to directly validate and refine the spatial models proposed here for ovarian endometriomas. Functionally validating the *CCL19*-*CCR7* and *NNMT* pathways in lesion-appropriate models, such as organoids cultured under niche-specific conditions, represents a critical next step for therapeutic development. Clinically, translating these insights requires the development of accessible biomarkers for lesion subtyping and prospective trials to test subtype-stratified treatment approaches. Sixth, a limitation of our study is the lack of direct comparison between healthy peritoneal and healthy ovarian tissues. The control group in our analysis consisted of healthy endometrium, which is the tissue of origin for both lesion types. However, the anatomical sites where EcP and EcO develop—the peritoneum and ovary—have distinct baseline transcriptional profiles that could influence the observed differences. Without a direct comparison of healthy peritoneum versus healthy ovary, we cannot definitively distinguish between disease-specific programs and tissue-of-origin effects. Future studies incorporating healthy peritoneal and ovarian tissues as additional controls would help clarify this distinction.

## Conclusion

Endometriosis has long been described as a disease with various manifestations. Our integrated analysis suggests a more nuanced reality: what we clinically term “endometriosis” actually encompasses at least two distinct disease entities with fundamentally different pathogenic mechanisms. The inflammatory *CCL19*/*CCR7*-driven peritoneal form and the structural *NNMT*-enriched ovarian form arise when susceptible endometrial cells encounter and adapt to different anatomical microenvironments. This niche-driven specification model, where EcP constructs chemokine-rich, immune-active ecosystems and EcO invests in fibromuscular differentiation and cellular survival programs, not only explains key clinical differences between lesion types but also provides a biologically grounded roadmap for precision therapeutic development. By recognizing and targeting the unique biology of each endometriosis subtype, we have moved closer to the goal of effective, personalized management for all patients affected by this complex and debilitating disorder.

## Supplementary Information

Supplementary Material 1.

Supplementary Material 2.

Supplementary Material 3.

Supplementary Material 4.

Supplementary Material 5.

Supplementary Material 6.

Supplementary Material 7.

## Data Availability

All the data analyzed in this study were available from public repositories. ScRNA-seq data are available under GEO accession number GSE179640. Spatial transcriptomic data are available under GEO accession GSE263897. Processed data and analysis codes will be made available in the GitHub repository upon publication.

## Abbreviations

EcP: Peritoneal endometriosis
EcO: Ovarian endometriosis
EuE: Eutopic endometrium
Ctrl: Control endometrium
scRNA-seq: Single-cell RNA sequencing
UMAP: Uniform Manifold Approximation and Projection
PCA: Principal Component Analysis
GO: Gene Ontology
CAF: Cancer-associated fibroblast
MDSC: Myeloid-derived suppressor cell
