# Supplementary figures and images for: Integrated single-cell and spatial transcriptomics reveal divergent immunological and stromal programs in peritoneal versus ovarian endometriosis
Source: BMC Womens Health. 2026 Apr 20;26:278. doi: 10.1186/s12905-026-04456-5 (PMC13224674; doi:10.1186/s12905-026-04456-5)

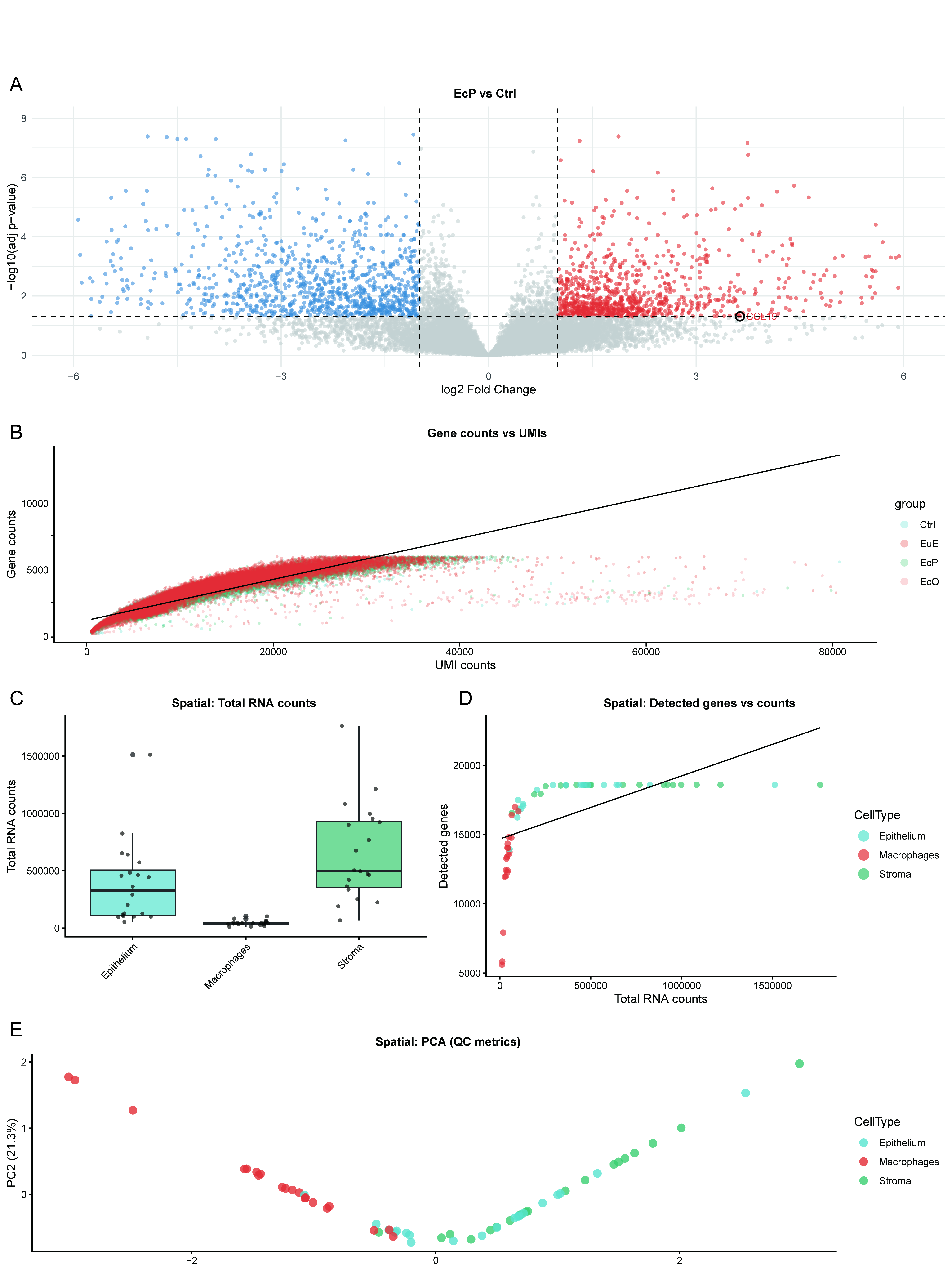

Supplement: Supplementary file 1 — Supplementary Material 1. [file 12905_2026_4456_MOESM1_ESM.tif]

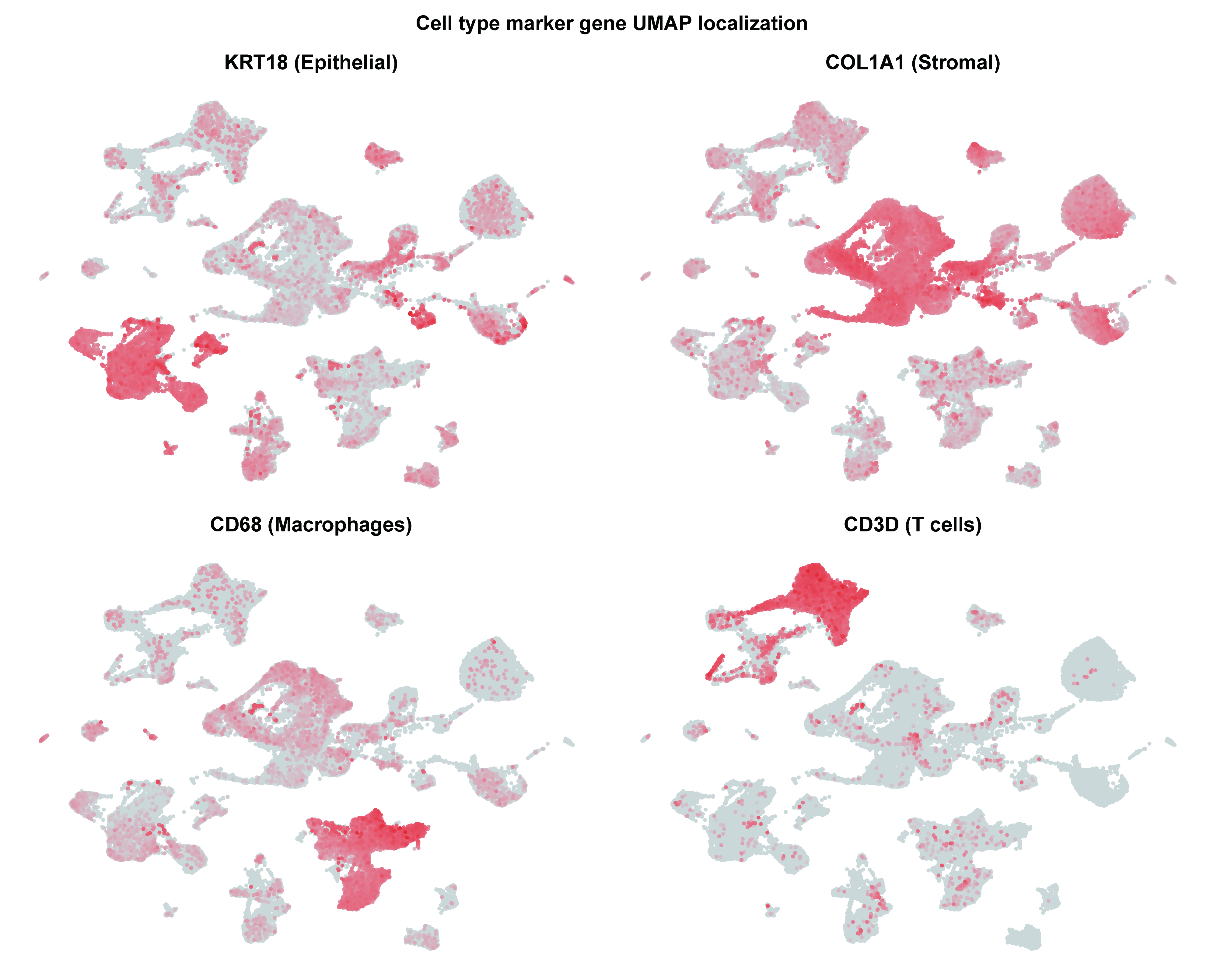

Supplement: Supplementary file 2 — Supplementary Material 2. [file 12905_2026_4456_MOESM2_ESM.tif]

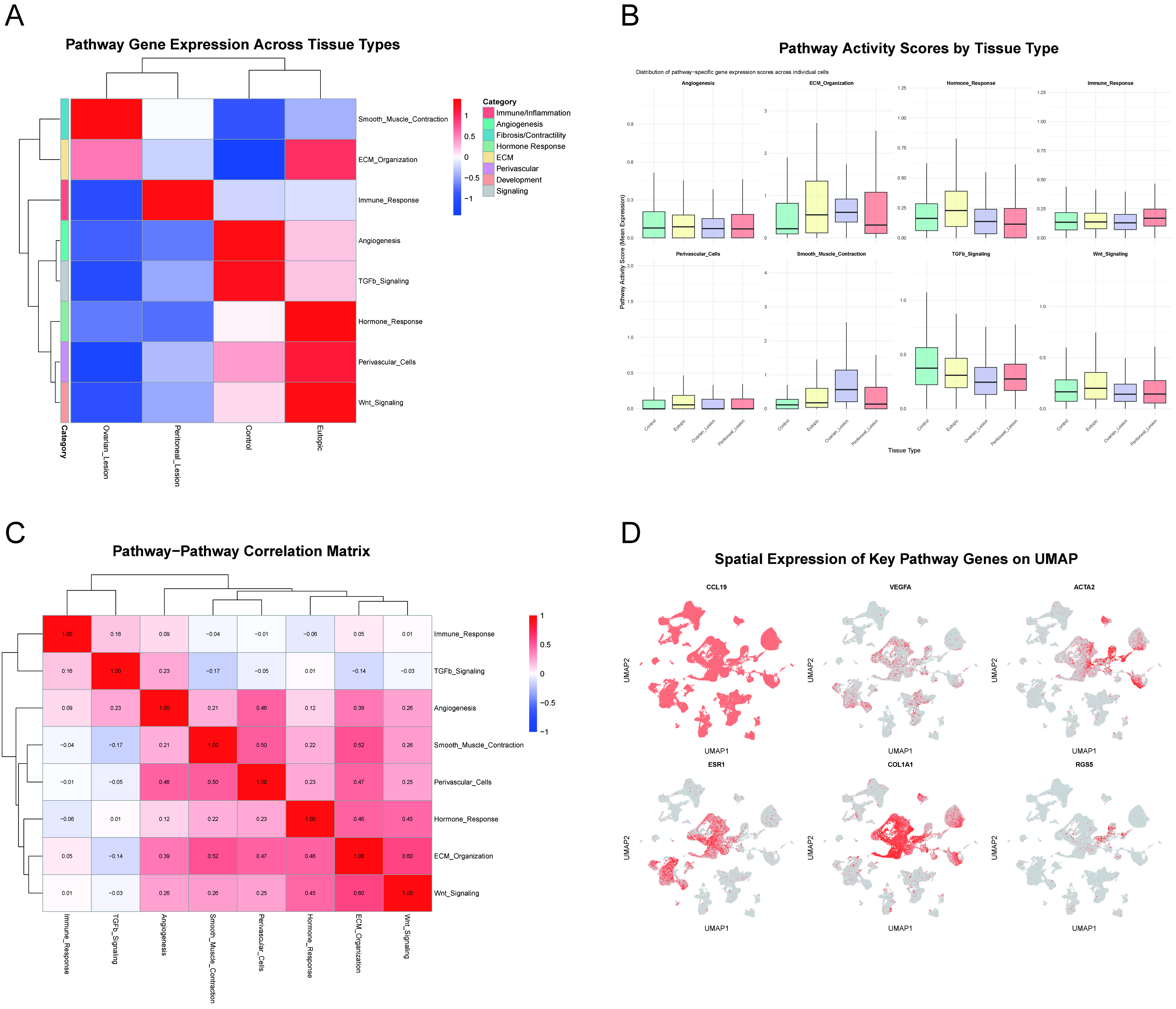

Supplement: Supplementary file 4 — Supplementary Material 4. [file 12905_2026_4456_MOESM4_ESM.tif]

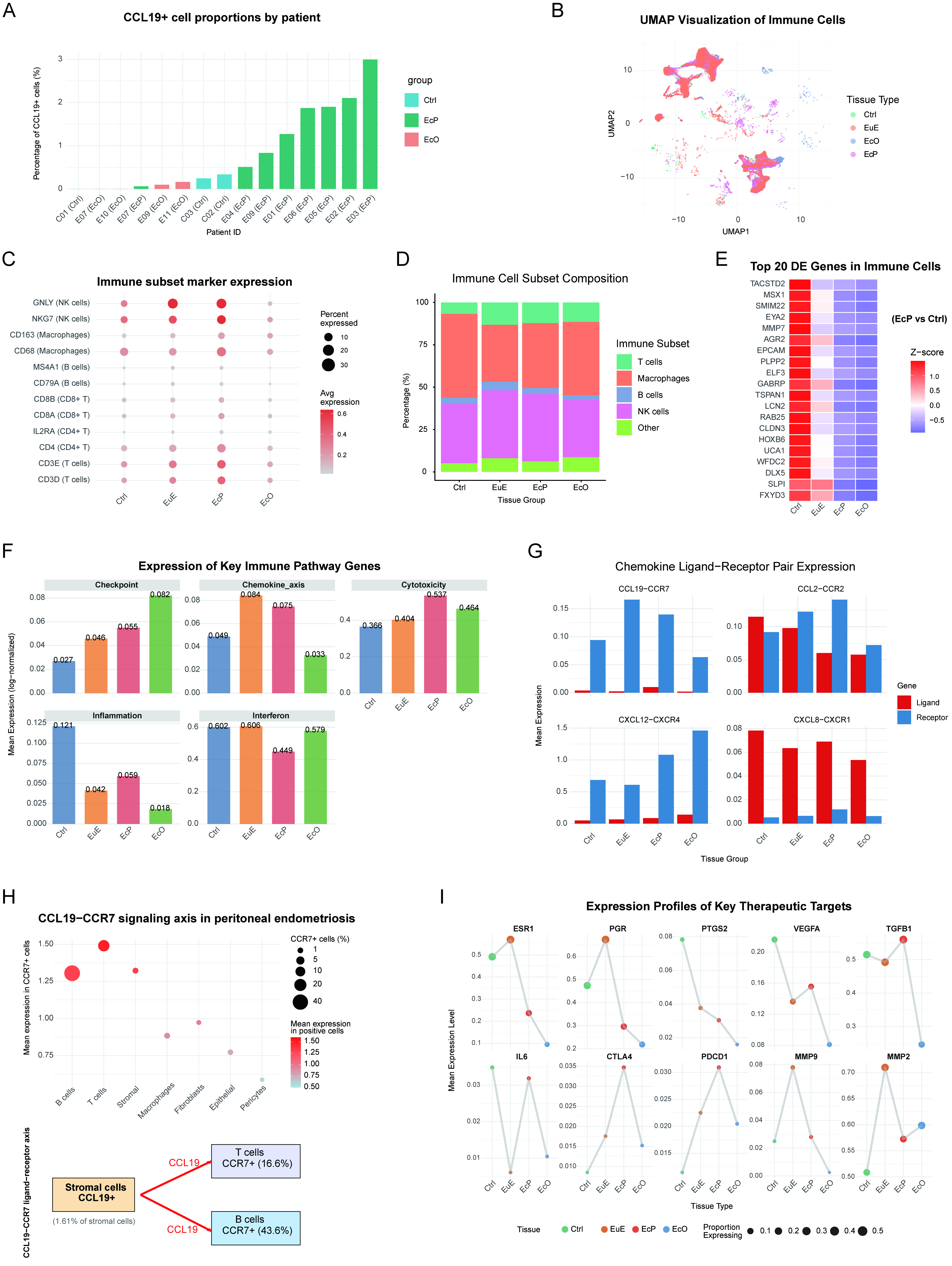

Supplement: Supplementary file 6 — Supplementary Material 6. [file 12905_2026_4456_MOESM6_ESM.tif]

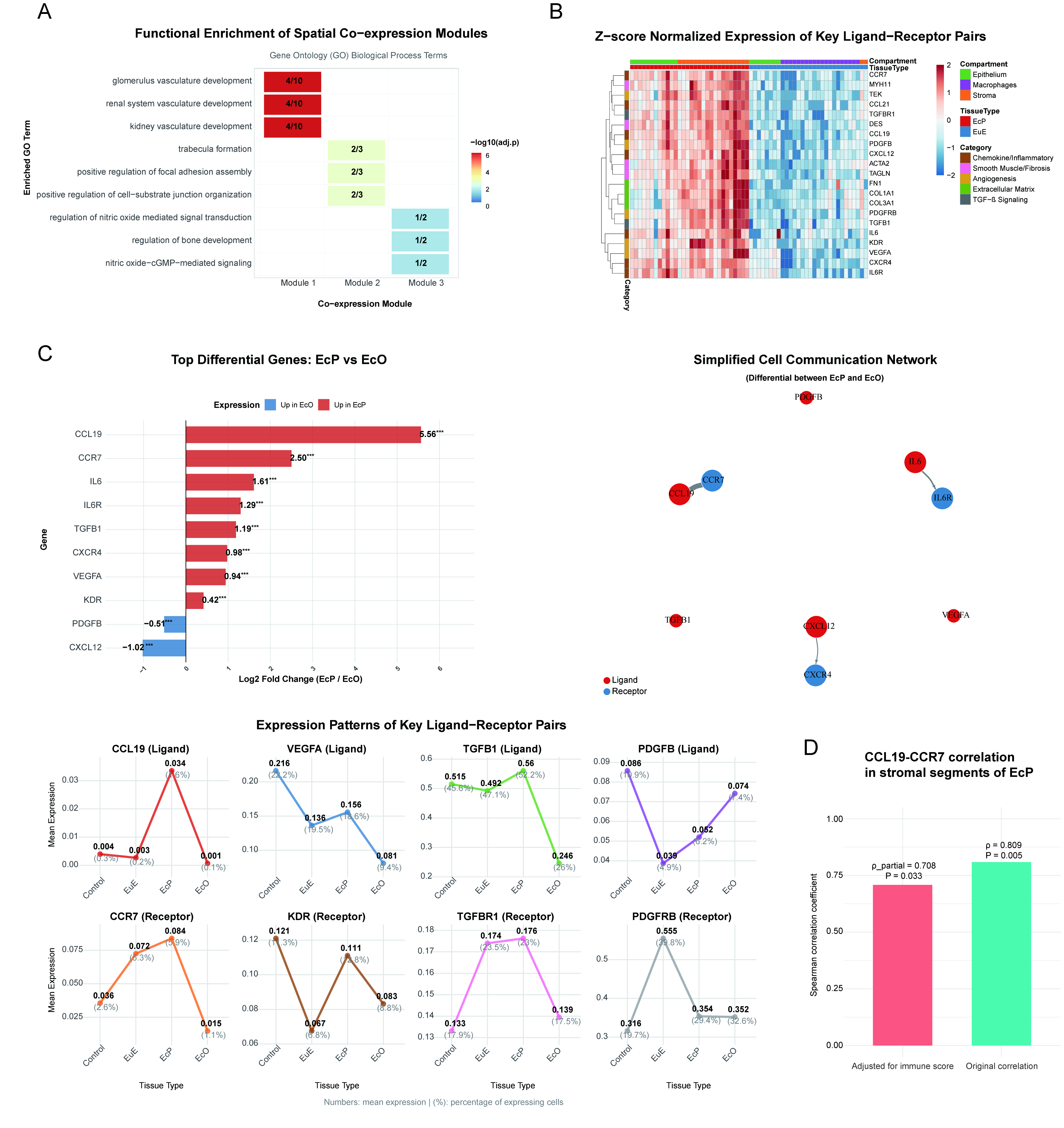

Supplement: Supplementary file 7 — Supplementary Material 7. [file 12905_2026_4456_MOESM7_ESM.tif]
