# Supplementary material for: Integrated single-cell and spatial transcriptomics reveal divergent immunological and stromal programs in peritoneal versus ovarian endometriosis
Source: BMC Womens Health. 2026 Apr 20;26:278. doi: 10.1186/s12905-026-04456-5 (PMC13224674; doi:10.1186/s12905-026-04456-5)
Supplement: Supplementary file 5 — Supplementary Material 5. [file 12905_2026_4456_MOESM5_ESM.docx]

**Supplementary data**

**Supplementary Figure 1.** Quality control metrics for single-cell and spatial transcriptomic data. (A) Violin plots showing distributions of quality control metrics across sample groups (Ctrl, EuE, EcP, EcO) for scRNA-seq data: number of genes detected per cell (left), UMI counts per cell (middle), and mitochondrial gene percentage (right). (B) Scatter plot showing the correlation between UMI counts and detected genes in scRNA-seq data, colored by sample group. The black line represents linear regression fit (Pearson r = 0.907, *P* = 2.2×10⁻¹⁶). (C) Box plots showing the distribution of total RNA counts in spatial transcriptomic segments, stratified by cellular compartment (Macrophages, Epithelium, Stroma). Each point represents an individual segment. (D) Scatter plot showing the correlation between total RNA counts and detected genes in spatial transcriptomic data, colored by compartment. The black line represents linear regression fit (Pearson r = 0.575, *P* = 1.58×10⁻⁶). (E) Principal component analysis (PCA) of spatial transcriptomic segments based on QC metrics, colored by compartment. Variance explained by each principal component is indicated in axis labels. Biological factors (tissue type and compartment) explained 60.9% of total variance, while technical batch effects accounted for only 12.3% (not shown).

**Supplementary Figure 2.** Cell type marker gene UMAP localization. UMAP feature plots showing the spatial distribution of representative cell type marker genes: *KRT18* (epithelial cells), *COL1A1* (stromal cells), *CD68* (macrophages), and *CD3D* (T cells). Color intensity represents expression level (light grey: low, red: high).

**Supplementary Figure 3.** Extended validation of differentially expressed genes. (A) Heatmap showing row-scaled expression of the top 30 differentially expressed genes (EcP vs Ctrl) across tissue types (Ctrl, EuE, EcP, EcO). Genes are clustered by expression similarity. Red indicates high relative expression, and blue indicates low relative expression. (B) UMAP feature plots showing expression distribution of non-core genes *MUC5B*, *LYVE1*, and *VEGFA*. Color intensity represents expression level (light grey: low, red: high). (C) ROC curves for *ESR1* and *PGR* distinguishing endometriotic lesions (EcP + EcO) from control endometrium. AUC values: *ESR1* = 0.666, *PGR* = 0.633.

**Supplementary Figure 4.** Comprehensive validation of pathway enrichment analyses in endometriosis. (A) Heatmap of z-score normalized expression for key pathway gene sets across four tissue types (Ctrl, EuE, EcP, EcO). Pathways are annotated by functional category (immune/inflammation, angiogenesis, fibrosis/contractility, hormone response, ECM organization, perivascular, development, signaling). (B) Box plots showing distribution of pathway activity scores across tissue types. Activity scores are calculated as the mean expression of pathway-specific genes per cell. Tissue sample sizes: Ctrl (n=10,351 cells), EuE (n=10,806), EcP (n=23,251), EcO (n=23,634). (C) Pathway-pathway correlation matrix. Color intensity indicates Pearson correlation strength (blue: negative, red: positive). Key correlations: Wnt signaling vs. ECM organization (r = 0.597), smooth muscle contraction vs. ECM organization (r = 0.521). (D) UMAP feature plots showing spatial distribution of representative pathway genes: *CCL19* (immune), *VEGFA* (angiogenesis), *ACTA2* (smooth muscle), *ESR1* (hormone), *COL1A1* (ECM), *RGS5* (perivascular). Color gradient indicates expression level.

**Supplementary Figure 5.** Extended immune profiling analysis. (A) Bar plot showing per-patient proportions of CCL19+ cells in Ctrl, EcP, and EcO. Each bar represents an individual patient. Color indicates tissue type. (B) UMAP visualization of immune cells across four tissue types. Colors indicate tissue of origin: Ctrl (blue, n=2,392 cells), EuE (green, n=8,095), EcP (red, n=11,073), EcO (purple, n=3,872). Immune cells identified by pan-immune marker (e.g., *PTPRC*) expression. (C) Dot plot showing expression of immune subset-specific markers across tissue types. Dot size represents the proportion of expressing cells; color intensity represents mean expression level. Markers include T cells (*CD3D*, *CD3E*), CD4+ T cells (*CD4*, *IL2RA*), CD8+ T cells (*CD8A*, *CD8B*), B cells (*CD79A*, *MS4A1*), macrophages (*CD68*, *CD163*), and NK cells (*NKG7*, *GNLY*). (D) Stacked bar chart showing the distribution of major immune subset across tissue types, based on lineage-specific markers assignment. (E) Heatmap showing expression of the top 20 differentially expressed genes in immune cells (EcP vs. Ctrl). Genes are row-scaled (z-score) and organized by hierarchical clustering. Color scale indicates expression level relative to mean (blue: low, red: high). (F) Bar plots showing mean expression of key immune pathway genes across tissue types. Pathways include immune checkpoint (*CTLA4*, *PDCD1*), chemokine axis (*CCL19*, *CCR7*), inflammation (*IL1B*, *IL6*), cytotoxicity (*GZMB*), and interferon response (*ISG15*). Error bars represent standard error of the mean. (G) Expression patterns of chemokine ligand-receptor pairs across tissue types. Bars represent mean expression of ligand (dark shade) and receptor (light shade) for each pair, highlighting preferential expression of the *CCL19*-*CCR7* axis in EcP. (H) Cellular basis of CCL19-CCR7 signaling in peritoneal endometriosis. (Upper) Dot plot showing CCR7 expression distribution across cell types. Dot size represents the percentage of CCR7+ cells; color intensity represents mean expression level in positive cells. (Bottom) Schematic model of the CCL19-CCR7 ligand-receptor axis, illustrating ligand source (CCL19+ stromal cells) and receptor-expressing cells (CCR7+ B cells and T cells). (I) Expression profiles of ten key therapeutic targets genes across tissue types. Points indicate mean expression; lines connect the same gene across groups. Point size represents the proportion of expressing cells.

**Supplementary Figure 6.** Extended spatial co-expression and intercellular communication analyses. (A) GO enrichment analysis of the three spatial co-expression modules. Heatmap displays the top enriched biological processes for each module, with tile color indicating significance (-log10 adjusted *P*-value) and text showing the proportion of module genes contributing to each term. Module 1 shows strong enrichment for renal vasculature development terms (adjusted *P* = 2.13×10⁻⁷), Module 2 for trabecula formation and focal adhesion processes (adjusted *P* = 1.48×10⁻³), and Module 3 for nitric oxide signaling and bone development regulation (adjusted *P* = 2.24×10⁻²). (B) Heatmap of z-score normalized expression for 21 key ligand-receptor pairs across 60 spatial segments from EcP and matched EuE. Samples are grouped by tissue type and compartment (macrophages, epithelium, stroma). (C) Multi-scale comparative analysis between endometriosis subtypes. (Upper left) Top differentially expressed genes between EcP and EcO. (Bottom) Expression patterns of four critical ligand-receptor pairs across tissue types. (Upper right) Differential gene interaction networks. (D) CCL19-CCR7 correlation in stromal segments of peritoneal lesions before and after adjustment for immune cell content. Left bar: partial correlation after controlling for immune cell score (ρ_partial = 0.708, P = 0.033). Right bar: original Spearman correlation (ρ = 0.809, P = 0.005), demonstrating that the association persists beyond immune cell contamination.
